# Effects of Prophylactic and Therapeutic Paracetamol Treatment during Vaccination on Hepatitis B Antibody Levels in Adults: Two Open-Label, Randomized Controlled Trials

**DOI:** 10.1371/journal.pone.0098175

**Published:** 2014-06-04

**Authors:** Anne M. C. M. Doedée, Greet J. Boland, Jeroen L. A. Pennings, Arja de Klerk, Guy A. M. Berbers, Fiona R. M. van der Klis, Hester E. de Melker, Henk van Loveren, Riny Janssen

**Affiliations:** 1 Centre for Health Protection, National Institute for Public Health and the Environment, Bilthoven, The Netherlands; 2 Department of Toxicogenomics, Maastricht University Medical Centre, Maastricht, The Netherlands; 3 Medical Microbiology and Virology, University Medical Centre Utrecht, Utrecht, The Netherlands; 4 Centre for Immunology of Infectious Diseases and Vaccines, National Institute for Public Health and the Environment, Bilthoven, The Netherlands; Copenhagen University Hospital Gentofte, Denmark

## Abstract

Worldwide, paracetamol is administered as a remedy for complaints that occur after vaccination. Recently published results indicate that paracetamol inhibits the vaccination response in infants when given prior to vaccination. The goal of this study was to establish whether paracetamol exerts similar effects in young adults. In addition, the effect of timing of paracetamol intake was investigated. In two randomized, controlled, open-label studies 496 healthy young adults were randomly assigned to three groups. The study groups received paracetamol for 24 hours starting at the time of (prophylactic use) - or 6 hours after (therapeutic use) the primary (0 month) and first booster (1 month) hepatitis B vaccination. The control group received no paracetamol. None of the participants used paracetamol around the second booster (6 months) vaccination. Anti-HBs levels were measured prior to and one month after the second booster vaccination on ADVIA Centaur XP. One month after the second booster vaccination, the anti-HBs level in the prophylactic paracetamol group was significantly lower (p = 0.048) than the level in the control group (4257 mIU/mL vs. 5768 mIU/mL). The anti-HBs level in the therapeutic paracetamol group (4958 mIU/mL) was not different (p = 0.34) from the level in the control group. Only prophylactic paracetamol treatment, and not therapeutic treatment, during vaccination has a negative influence on the antibody concentration after hepatitis B vaccination in adults. These findings prompt to consider therapeutic instead of prophylactic treatment to ensure maximal vaccination efficacy and retain the possibility to treat pain and fever after vaccination.

**Trial Registration:**

Controlled-Trials.com ISRCTN03576945

## Introduction

Paracetamol (acetaminophen) is an analgesic and antipyretic drug widely used in children and adults [1]. In many European countries, paracetamol is used prophylactically (preventative treatment) to reduce pain and fever associated with vaccination [2]. In the Netherlands, the advice is to be cautious with the use of paracetamol during vaccination and only children who experienced fever or persistent screaming after vaccination are advised to use paracetamol prior to further vaccinations [3]. Despite this advice to restrict paracetamol to those children with previous reactions, many parents give paracetamol to their children prior to or just after the vaccine administrations. Estimates of paracetamol use made by the Dutch National Immunization Program (NIP) range from 19–27% prophylactically, and up to 49% prophylactically and therapeutically [4]. Data on use of paracetamol by adults during vaccination are not available.

Paracetamol is generally regarded as safe, besides the hepatotoxic effects at higher doses, and is abundantly used as an over the counter drug [5]. Paracetamol was long considered to be a drug without anti-inflammatory effects and the immunomodulatory properties of paracetamol have only recently been described. Paracetamol suppressed several immune parameters in animal studies, such as T-cell dependent antibody response [6], [7]. Toxicogenomic studies revealed an influence on gene expression in lymphocytes consistent with inhibition of cell proliferation of immune cells [8]. This information is in line with older studies that suggested inhibitory effects on clearance of chickenpox in children and rhinovirus [9], [10]. Furthermore, several epidemiologic studies suggested an association between paracetamol use in children and adults and development of asthma. Asthma is a disease characterized by deregulated inflammatory responses and possible interference of paracetamol with these immune processes was proposed to underlie this association.[11]–[14].

Recently published results suggest a negative influence on vaccination response, i.e. a decrease in antibody levels, in infants who received paracetamol prior to vaccination (ten-valent pneumococcal vaccine) in order to prevent fever [15]. The decreased antibody levels were only observed after paracetamol treatment prior to the first vaccination, the priming of the immune response, and not when paracetamol was given prior to booster immunisations.

The potential immunosuppressive effects of paracetamol that were recently shown to affect the efficacy of vaccination could result in increased susceptibility to infections, especially when paracetamol is applied shortly before active immune processes are induced. To date, it has not been studied whether use of paracetamol also affects the immune response after a primary vaccination in adults. In addition, it is not known to what extent timing of paracetamol intake, i.e. prophylactic or therapeutic, affects the response. The present study was performed to investigate possible effects of prophylactic and therapeutic paracetamol use in adolescents, on the quantitative antibody response to hepatitis B vaccination. Effects of paracetamol are highly relevant for health authorities who advise on the use of paracetamol as a treatment for vaccination-induced adverse responses.

## Methods

### Study Design

The present study was composed of two phases. First a pilot study (phase 1) was performed to investigate effects of prophylactic use of paracetamol on the immune response in adults after a hepatitis B vaccination. Thereafter, phase 2 was performed to confirm prophylactic effects of paracetamol found in phase 1, and to evaluate the effect of therapeutic use of paracetamol on the immune response to hepatitis B vaccination. The two (phase 1 and phase 2) randomized, controlled, open-label studies were performed at the Hogeschool of Utrecht from October 3, 2011 to April 20, 2012 (phase 1), and from October 8, 2012 to April 26, 2013 (phase 2).

Both studies were undertaken according to Good Clinical Practice and the Declaration of Helsinki (Somerset West, 1996 version). The protocol (NL36577.041.11) was approved by the ethics review committee of UMC Utrecht, and is available as supporting information as well as the CONSORT checklist; see Protocol S1 and Checklist S1. The study was registered at the European Clinical Trials database (EudraCT number: 2011–000923–33) prior to recruitment of participants. Due to a communication error this study was registered at the ISRCTN register (ISRCTN03576945) after start of recruitment. The authors confirm that all ongoing and related trials for this drug/intervention are registered.

### Study Population

**Figure 1:**
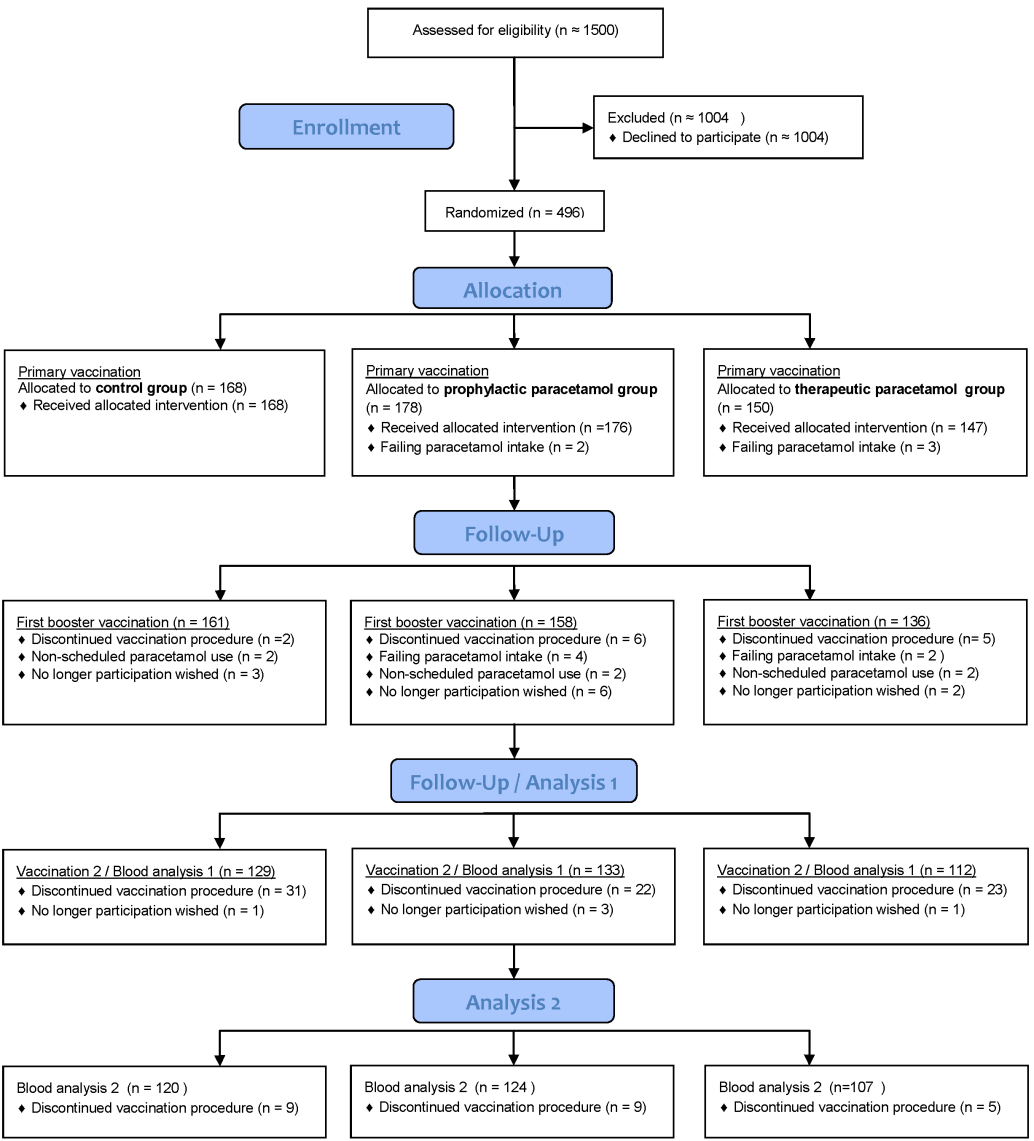
Consort 2010 Flow Diagram. The participation flow during the vaccination, intervention and blood collection.

Study participants were healthy young health care students of 18 years or older, who are routinely vaccinated against hepatitis B. The students were approached by the Hogeschool Utrecht for a hepatitis B vaccination and at the same time participation in the study was offered, as described in the Consort 2010 Flow Diagram (Fig. 1). Students were enrolled in the study after written informed consent was obtained. Participants were not included in case they used NSAIDs or paracetamol within 48 hours before the first vaccination; or if they had a history of acute or chronic hepatitis B; earlier vaccination against hepatitis B, medical immunosuppressive treatment, primary or secondary immunodeficiency, or allergic reactions to components of the hepatitis B vaccination or paracetamol. In case participants had fever (>38.5°C), vaccination or blood collection were postponed. Participants that did not take paracetamol according to the protocol they were assigned to, were excluded during the study and blood was not collected. Therefore, the analysis was a per-protocol analysis.

### Intervention

Hepatitis B vaccines (Engerix-B) were manufactured by GlaxoSmithKline Biologicals (GSK), Rixensart, Belgium. Hepatitis B vaccines contained 20 µg recombinant hepatitis B surface antigen (HBsAg). Vaccines were administered intramuscularly into the right or left deltoid muscle into a strict schedule at 0 (primary vaccination), 1 month (5 weeks±4 days; first booster vaccination) and 6 months (23 weeks±4 days; second booster vaccination).

**Figure 2:**
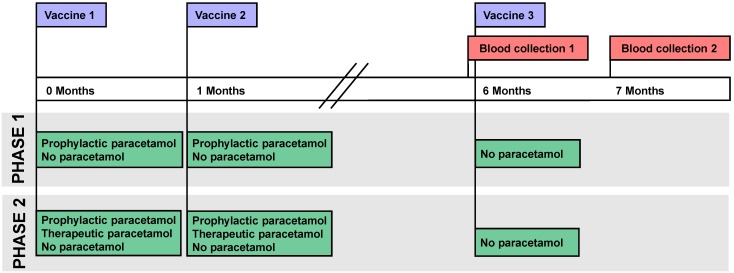
Timeline of the vaccination, intervention and blood collection. The study was composed of two phases. In phase 1, one prophylactic and one control group were investigated. In phase 2, one prophylactic paracetamol, one therapeutic paracetamol and one control group were investigated. The paracetamol groups received paracetamol after the primary and first booster hepatitis B vaccine doses, at 0 and 1 month. Participants received no paracetamol after the second booster vaccine dose at 6 months. The control group received no paracetamol or placebo during the hepatitis B vaccination procedure. Blood (14–21 mL) was taken of all participants prior to and one month after the second booster vaccination.

**Figure 3:**
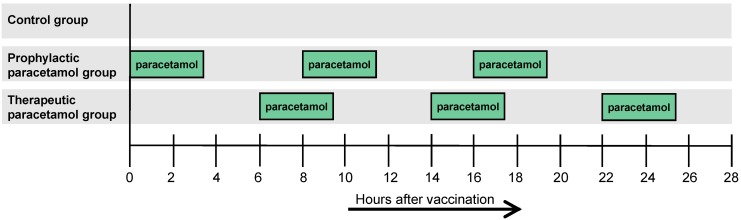
Timeline of the paracetamol treatment after the primary and first booster vaccination. The prophylactic paracetamol group received three doses of paracetamol (1000 mg/dose) starting immediately after vaccination, 8 hours and 16 hours after the vaccination. The therapeutic paracetamol group received three doses of paracetamol (1000 mg/dose) starting 6 hours after vaccination, 14 hours and 22 hours after vaccination. The control group received no paracetamol or placebo during the hepatitis B vaccination procedure.

The timeline of the vaccination, intervention and blood collection in phase 1 and 2 is shown in figure 2. In phase 1 the study participants were randomly assigned (IBM SPSS Statistics version 19) to two groups (1∶1): prophylactic paracetamol group and control group. In phase 2 the study participants were randomly assigned to three groups (1∶2∶1): prophylactic paracetamol, therapeutic paracetamol and control group. The paracetamol treatment consisted of three doses of paracetamol purchased from Omega Pharma (The Netherlands) administrated orally within the first 24 hours directly (prophylactic; t = 0 h, t = 8 h, and t = 16 h) or 6 hours after (therapeutic; t = 6 h, t = 14 h, and t = 22 h) the primary and first booster vaccination (Fig. 3). The first administration of paracetamol in the prophylactic group was performed immediately after vaccination in the vaccination clinic. The second and third administrations were done at home every 8 h. The therapeutic group took all the administrations at home every 8 h.

The dose of paracetamol was the maximal allowed paracetamol dose per administration (1000 mg/8 hours) for adults. The control group received no paracetamol or placebo. None of the participants received paracetamol after the booster vaccine dose at 6 months. Furthermore, participants were asked to restrain from use of more paracetamol or NSAIDs 48 hours after the vaccination in the treated and control groups. Participants that reported paracetamol use other than that prescribed in this study were excluded.

Blood samples (14 ml) were collected prior to and one month after the second booster vaccination in blood collection tubes (Ref#367955, Becton Dickinson, US).

### Laboratory Methods

Qualitative and quantitative HBsAg antibody levels in the sera were measured on the ADVIA Centaur XP system (Siemens Healthcare Diagnostics Inc., USA) by using the ADVIA Centaur Anti-HBs assay according to the manufacturer’s protocol. In short, 100 µl sample was added to 100 µl of inactivated human HBsAg (subtype ad and ay, about 2 µg/ml) coupled to magnetic latex particles (solid phase) in a cuvette. Then 50 µl inactivated HBsAg labeled with acridinium ester was added. After 7.5 minutes of incubation at 37°C, the cuvette was washed with PBS. Chemiluminescence was initiated by adding 300 µl acid reagents and 300 µl base reagents were added. Antibody levels were measured as International Units per Liter (IU/L). Values above the 10.0 IU/L were considered to be positive and give sufficient protection against hepatitis B, according to the World Health Organization.

Measurement of the anti-HBs level prior to the second booster vaccination was used to exclude participants that had an earlier vaccination against hepatitis B. Participants with values above the 10,000 IU/L were asked for all their vaccination certificates to control the hepatitis B vaccination status, and, if necessary, these participants were excluded from further participation in this study.

### Statistical Analysis

The power calculation is based on the Z-test for the difference between expected values of log10 concentrations in two groups, taking into account the different variance in the two groups. A ratio of 1.65 was used, as the significant effects of the pneumococcal antibody concentrations in the Prymula study gave this average ratio [15]. More details of the power calculation are available as supporting information; see Protocol S1.

The effect of paracetamol on the vaccination response was evaluated in two different manners; the number of participants whose antibody levels were above the protected level (10 IU/L), and the geometric mean concentrations (GMCs) of the anti-HBs. GMCs of the anti-HBs levels with 95% confidence interval (CI) were calculated for the control, prophylactic paracetamol and therapeutic paracetamol group. The two phases of the present study were performed according to the same protocol, same inclusion and exclusion criteria and corresponding time between the vaccine administrations and blood collections. Therefore, the data of phase 1 and phase 2 have been combined into one dataset (supporting information; see Table S1) to increase the power of the study. Small year differences between the two phases were adjusted by correcting for the difference between the median anti-HBs levels of the phase 1 and phase 2 control group; the prophylactic and therapeutic groups were adjusted with the same value correction as their corresponding control group. The GMCs of the anti-HBs levels of phase 1 and phase 2 before and after correction are presented in the supporting information; see Table S2. Further analyses were performed on the combined dataset.

Statistical differences were calculated using the Mann–Whitney test, as the data were not normally distributed. Based on the study of Prymula et al., 2009 it was expected that the paracetamol groups would have a lower antibody concentration, as significant decreasing effects of paracetamol on the vaccination response on pneumococcal and tetanus antibody levels were found. Although, the effect of paracetamol on hepatitis B was not significantly different, a decrease in antibody levels was still apparent. These effects point in the same direction, lower antibody concentration after paracetamol use during vaccination. Therefore, an unpaired, one-sided test was performed.[15].

Statistical differences in drop-out percentages were calculated using the Chi-Square test. A P-value of <0.05 was considered statistically significant.

## Results

### Study Participants

The numbers of participants and their characteristics in the different groups of phase 1 and phase 2 studies are shown in table 1 and in the Consort Flow Diagram (Fig. 1). 177 participants were enrolled and vaccinated in phase 1 (pilot study) and 319 participants in phase 2. Study participants were randomly divided in a control, prophylactic or therapeutic paracetamol group.

**Table 1:** Number of participants and characteristics during phase 1 and phase 2.

	Paracetamol Treatment	Primary Vaccination(N = )	First boostervaccination (N = )	Second booster vaccination/Blood collection 1 (N = )	Blood collection 2 (N = )	Drop-out(%)	Age range(years)	Male (%)
Phase 1	Control	85	83	67	65	23.5	18–45	29.2
	Prophylactic	92	88	77	71	22.8	18–25	33.8
	Total	177	171	144	136	23.2	18–45	31.6
Phase 2	Control	83	78	64	59	28.9	18–48	27.1
	Prophylactic	86	70	52	49	43.0	18–32	30.6
	Therapeutic	150	136	112	107	28.7	18–47	33.6
	Total	319	284	238	215	32.6	18–48	31.2
Phase 1+2	Control	168	161	133	124	26.2	18–48	28.2
	Prophylactic	178	158	129	120	32.6	18–32	32.5
	Therapeutic	150	136	112	107	28.7	18–47	33.6
	Total	496	455	374	351	29.2	18–48	31.3

Overall there was a drop-out percentage of 27.6% (23.2% in phase 1 and 32.0% in phase 2). The drop-out percentage was not significantly different (p = 0.4187) between the control, prophylactic and paracetamol group. Dropping out occurred for several reasons; students that discontinued their education and therefore stopped the vaccination procedure or students that stopped the vaccination procedure without stating reasons (n = 112); students that reported paracetamol use, unrelated to the present study, in the 48 hours prior to or after the booster vaccination (n = 6); students that forget to take a dose of paracetamol at the right time (n = 11); students that no longer wished to participate in the present study (n = 16).

The mean age of the vaccinated cohort at the time of the primary vaccination was 21.09 years with a minimal age of 18 years and a maximal age of 48 years. The distribution of the age of the study cohort was divided in typical student ages (18–25 years) and individuals with ages scattered up to 48 years. Therefore an outlier test based on age was performed. The outcome of this test was 25.575 years and this number was round up to 26 years. Only 5.4% of the study participants were 26 years or older (median = 20.05, min-max = 18.03–48.56). No significant differences were found in the anti-HBs levels of the students ≥26 years compared to the anti-HBs levels of the students <26 years.

The vaccinated cohort comprised 68.7% female and 31.3% male participants; the gender distribution was similar and not significantly different in the control, prophylactic and therapeutic paracetamol group. There were no significant differences in anti-HBs levels between female and male participants.

Anti-HBs levels were not statistically different for female and male participants and between different age groups (<26 years and ≥26 years). Therefore, further data analysis of the anti-HBs levels between study groups was based on data of all participants and performed without age and gender as confounding factors.

### Anti-HBs Levels

**Figure 4:**
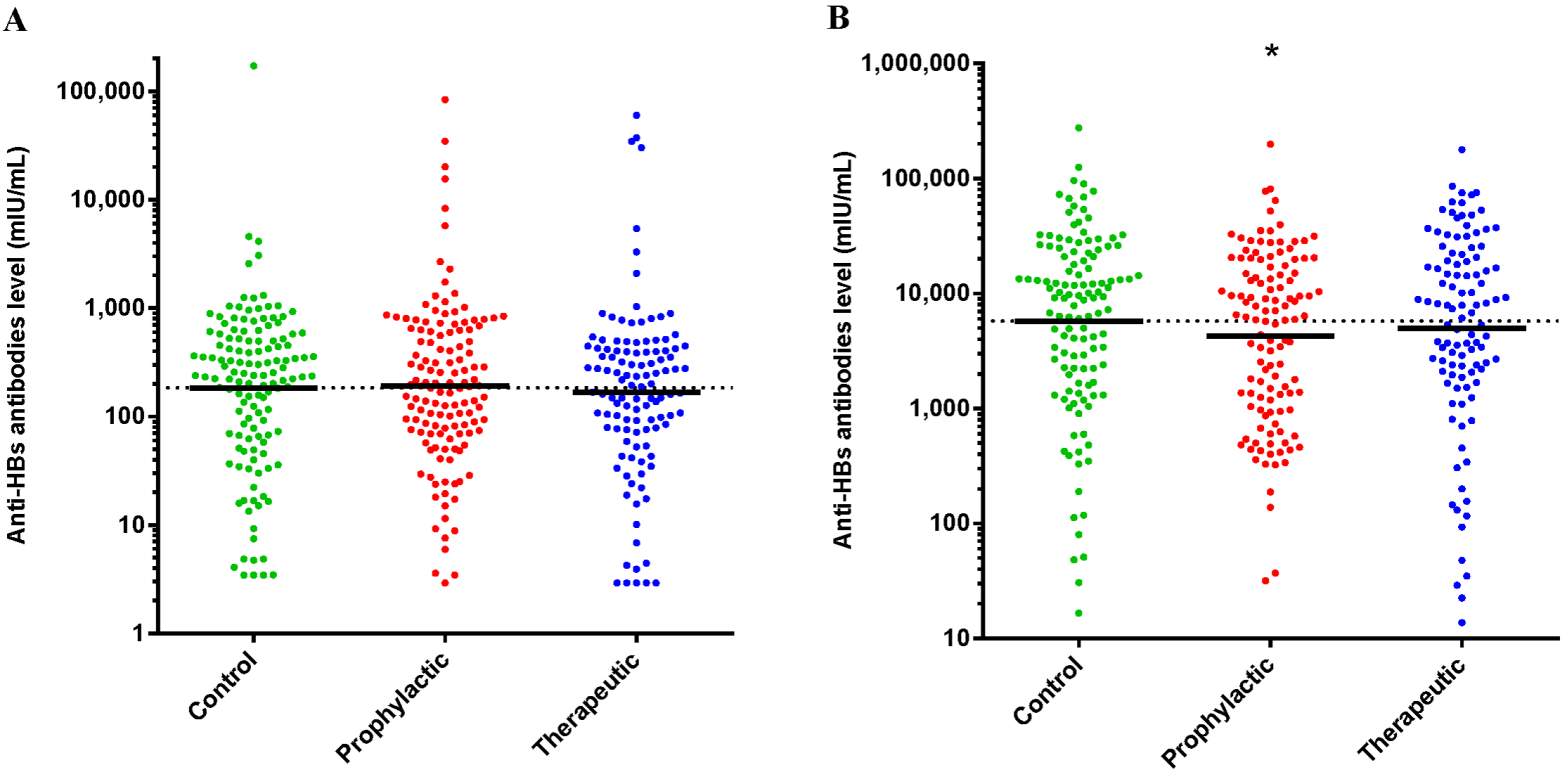
Anti-HBs levels prior (A) to and one month after (B) the second booster vaccination. Line at GMC. A) The anti-HBs levels in the groups were not significantly different. B) The anti-HBs level in the prophylactic paracetamol group (4257 mIU/mL) is significantly lower (p = 0.048) from the anti-HBs level in the control group (5768 mIU/mL). The anti-HBs level in the therapeutic paracetamol group (4958 mIU/mL) was not significantly different from the control group. Significant differences in anti-HBs levels were tested by using the Mann-Whitney U test. * = p<0.05.

Anti-HBs levels were measured just before and one month after the second booster vaccination. Before the second booster no differences were observed between the groups (Fig. 4, panel A). After completion of the study. i.e. one month after the second booster hepatitis B vaccination, none of the students displayed a response below the protection level. Subsequently, we focused on comparison of the GMC of the different treatment groups. The anti-HBs level in the prophylactic paracetamol group was significantly lower (p = 0.048) than the level in the control group (Fig. 4, panel B) by using the Mann-Whitney U test. The reduction in anti-HBs level in the prophylactic paracetamol group was 26% as compared to the control group (4257 mIU/mL vs. 5768 mIU/mL) indicating that paracetamol decreases the induction of antibody response in adults. The anti-HBs level in the therapeutic paracetamol group was not different (p = 0.34) from the level in the control group (4958 mIU/mL vs. 5768 mIU/ml) indicating the timing of paracetamol determines its immunomodulatory effect.

**Figure 5:**
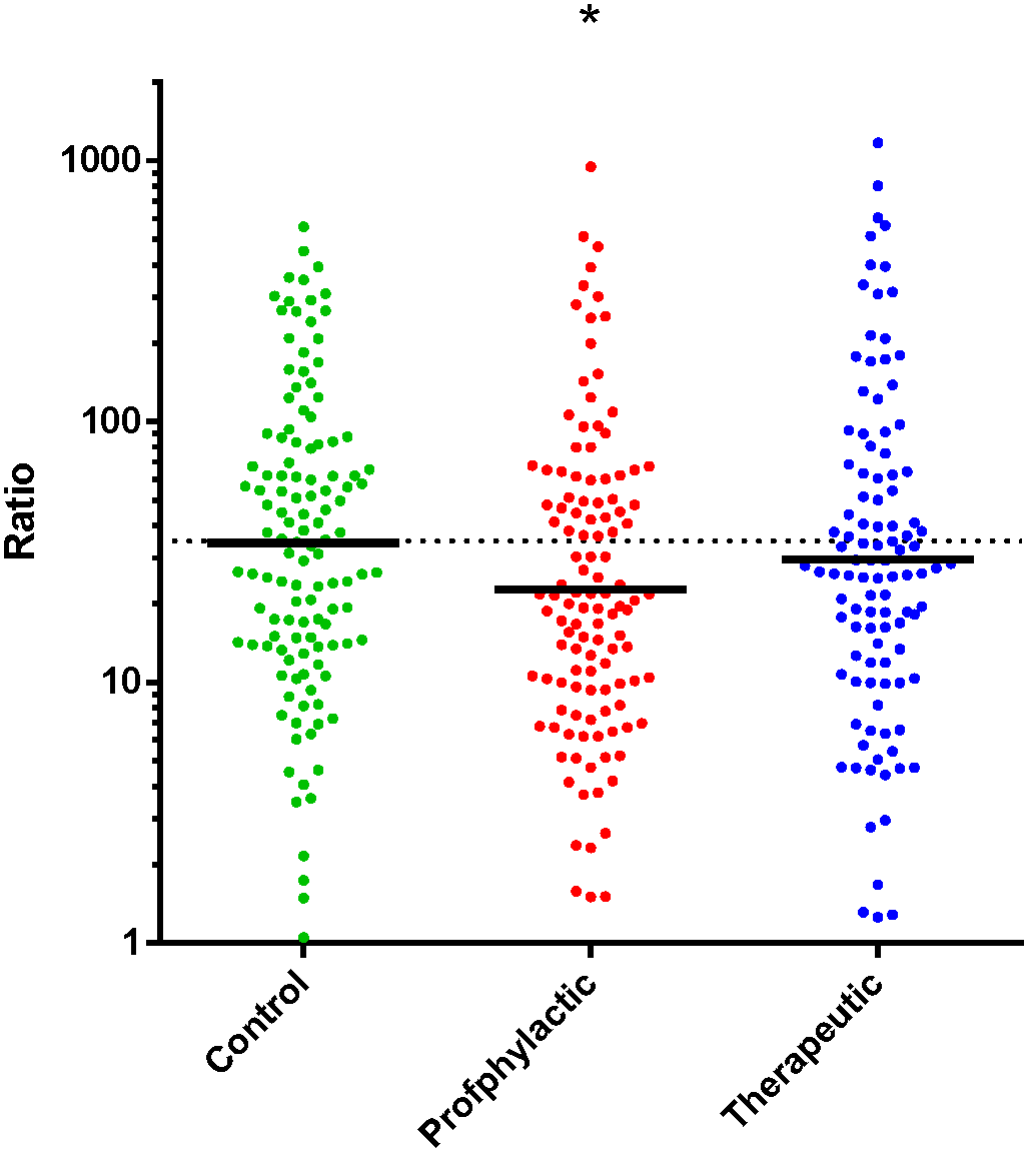
Ratio between the anti-HBs levels prior to and one month after the second booster vaccination. The increase in antibody concentration after the second booster vaccination is expressed in ratio. The ratio in the prophylactic paracetamol group (20.3) is significantly lower from the ratio in the control group (34.9). The ratio in the therapeutic paracetamol group (27.4) was not significantly different from the control group. * = p<0.01.

Furthermore, the increase in individual antibody concentration after the second booster vaccination was calculated and expressed as the ratio of the two (Fig. 5). The ratio in the prophylactic paracetamol group is significantly lower (p = 0.005) from the ratio in the control group. The ratio in the therapeutic paracetamol group was not different from the ratio in the control group (p = 0.17). These results indicate that only prophylactic paracetamol treatment during the primary vaccinations affects the initial immune response resulting in a diminished effect of the second booster vaccination.

## Discussion

Our study showed, in accord with earlier studies published by Prymula et al. [15], that exposure to paracetamol can suppress immune function to antigens derived from bacterial and viral pathogens, and this might have consequences for resistance to infectious agents. The effects noted were modest, but modest suppression on a population basis may have considerable consequences as has been noted with exposure to environmental pollutants inducing similar levels of immune suppression [16]–[18].

The effects of prophylactic and therapeutic paracetamol treatment were observed in a human model in which specific antibody responses to hepatitis B antigen were assessed. The main finding of this study is that prophylactic use of paracetamol exerts a negative effect on the primary antibody response after hepatitis B vaccination in adults. In addition, we show that such an inhibitory effect is not observed when paracetamol is given therapeutically. Apparently, the timing of paracetamol determines its immunomodulatory effects.

Our finding extends the data recently published by Prymula et al. that showed that prophylactic use of paracetamol inhibited the induction of antibodies to a combination of child-hood vaccines in infants [15]. Infants were vaccinated with the hexavalent diphtheria-tetanus-3-component acellular pertussis-hepatitis B-inactivated poliovirus types 1, 2, and 3-H influenza type b and oral human rotavirus vaccines. Prymula et al. aimed to study the effect of paracetamol use on the occurrence of side-effects to vaccination. While pain and fever were reduced, an inhibitory effect of paracetamol on the induction of antibodies to these vaccines was observed. The decrease in GMC that was observed was approximately thirty-five percent. In the present study a decrease of twenty-six percent was measured in the prophylactic paracetamol group indicating that the effects of paracetamol in adults is equivalent to those in infants. In contrast to the inhibitory effects of prophylactic paracetamol treatment on antibody responses, paracetamol did not exert similar significant effects when given therapeutically. Nevertheless, a slightly non-significant decrease in antibody response was visible in the therapeutic paracetamol group compared to the control group. To our knowledge our study is the first in which the effects of prophylactic and therapeutic paracetamol treatment on response to vaccination were compared.

The antibody levels of the control and both paracetamol groups display large variation, reflecting the variation in the human vaccine responses and thus the unique immune response of every individual after vaccination and the various physical and genetic factors that influence the response to vaccination [19], [20]. Although the antibody concentrations in this study were significantly lower in the prophylactic group, all study participants were considered to be protected against hepatitis B after the full schedule since all titers were above 10.0 IU/L, the threshold for protection against hepatitis B, according to the World Health Organization [21]. Since anti-HBs levels are measured quantitative and anti-HBs levels are standardized against an international reference and expressed in International Units, hepatitis B vaccination is a good model to investigate the effects of paracetamol on immune responses after vaccination in a safe manner. However, there is clear evidence that paracetamol has an effect on the functionality of the immune system, which could be relevant under conditions when the immune response to either vaccination or pathogen is already suboptimal.

However, the suppressive effect of paracetamol was not found in a comparable study with influenza vaccinations in elderly people [22]. Participants in the influenza study were many times exposed during their life to influenza viruses before vaccination, whereas infants had their primary contact with the viruses/bacteria vaccine antigens in the study of Prymula et al. Participants of the present study also encountered hepatitis B antigens for the first time. These results indicate that inhibitory effects of paracetamol are especially evident during the vaccinations that prime the immune response.

The development of vaccine antigen-specific memory after vaccination is a slow multistep process taking several days. The effects of paracetamol on the vaccination response in adults found in this study suggest that paracetamol plays an important role in the first six hours after the primary vaccinations. This is exemplified by our observation that the vaccination response was only significantly decreased in the prophylactic paracetamol treatment group and not in the therapeutic treatment group. The finding that paracetamol influences the vaccine response only when given directly at the time of vaccination, point to an effect during the early stages of the immune response, when APC-T cell interaction take place in lymphatic tissues [23]–[25]. Paracetamol may decrease the amount of glutathione thereby impairing respiratory antioxidant defenses [26]. Decreased glutathione levels could influence lymphocyte activation mechanisms [27], [28]. Another potential mechanism is the capacity of paracetamol to suppress fever by influencing the COX-2 activity and the production of prostaglandin E_2_ that stimulate the accompanying immune cell recruitment [29]. It is tempting to speculate that decreased recruitment of APC to the site of vaccination, and decreased activation of these cells during the early stages of induction of immunity underlie our observation, a mechanism which was already previously proposed by Prymula et al. although their study only included a prophylactic treatment group.

In the present study, we showed that prophylactic paracetamol treatment during vaccination has a negative influence on the antibody concentration after hepatitis B vaccination in adults. In the Netherlands, paracetamol is mostly used therapeutically after vaccination when side effects start to occur. Since therapeutic paracetamol treatment starting 6 hours after vaccination did not significantly inhibit the response to vaccination, our data indicate that treatment of fever and pain may be without disadvantages. These results are not only important for hepatitis B vaccination procedures, but also for other primary vaccinations in the national immunization program, although we cannot exclude effects of therapeutic paracetamol treatment on vaccines other than hepatitis B.

Most important, these findings prompt to consider therapeutic instead of prophylactic treatment to ensure maximal vaccination efficacy and retain the possibility to treat pain and fever after vaccination.

## Supporting Information

Table S1 **Anti-HBs levels of all participants.**
(DOCX)

Table S2 **Geometric concentrations (GMC) with 95% conficence intervals of the treatment groups.**
(DOCX)

Checklist S1 **CONSORT Checklist.**
(DOC)

Protocol S1 **Trial Protocol.**
(DOC)
