# Supplementary material for: Effects of Prophylactic and Therapeutic Paracetamol Treatment during Vaccination on Hepatitis B Antibody Levels in Adults: Two Open-Label, Randomized Controlled Trials
Source: PLoS One. 2014 Jun 4;9(6):e98175. doi: 10.1371/journal.pone.0098175 (PMC4045752; doi:10.1371/journal.pone.0098175)
Supplement: Table S1 — Anti-HBs levels of all participants. (DOCX) [file pone.0098175.s001.docx]

**Table S1. Anti-HBs levels of all participants**

| **Code** | **Phase** | **Treatment** | **Gender** | **Age** | **Anti-HBs level 1***  **(IU/L)** | **Anti-HBs level 2***  **(IU/L)** |
| --- | --- | --- | --- | --- | --- | --- |
| V458 | 1 | Control | Female | <26 | 3.1 | 45.7 |
| X402 | 1 | Control | Male | <26 | 13.5 | 106.6 |
| Y205 | 1 | Control | Female | >26 | 60.9 | 112.1 |
| P123 | 1 | Control | Female | <26 | 6.7 | 312 |
| P761 | 1 | Control | Female | <26 | 12.03 | 330.7 |
| L113 | 1 | Control | Male | <26 | 35.96 | 402 |
| B792 | 1 | Control | Female | <26 | 200.14 | 457 |
| X803 | 1 | Control | Female | <26 | 349.96 | 551 |
| M216 | 1 | Control | Female | <26 | 62.8 | 853 |
| Q837 | 1 | Control | Male | >26 | 4.34 | 956 |
| U792 | 1 | Control | Female | <26 | 134.12 | 989 |
| T320 | 1 | Control | Male | <26 | 60.23 | 1230 |
| V562 | 1 | Control | Female | <26 | 70.36 | 1243 |
| H358 | 1 | Control | Male | <26 | 3.1 | 1283 |
| U729 | 1 | Control | Male | <26 | 32.97 | 1600 |
| N560 | 1 | Control | Female | >26 | 27.07 | 1867 |
| L908 | 1 | Control | Female | <26 | 29.6 | 2104 |
| P281 | 1 | Control | Female | <26 | 444.19 | 2122 |
| A618 | 1 | Control | Female | <26 | 15.03 | 2148 |
| Y268 | 1 | Control | Male | <26 | 187.3 | 2707 |
| W242 | 1 | Control | Female | <26 | 104.16 | 2897 |
| M650 | 1 | Control | Female | <26 | 862 | 3162 |
| P390 | 1 | Control | Female | <26 | 215.78 | 3874 |
| Z826 | 1 | Control | Male | <26 | 145.38 | 3995 |
| L907 | 1 | Control | Female | <26 | 281.73 | 4236 |
| R636 | 1 | Control | Male | <26 | 16.41 | 4626 |
| Z850 | 1 | Control | Female | <26 | 648 | 4736 |
| T340 | 1 | Control | Female | <26 | 379.36 | 5933 |
| J776 | 1 | Control | Male | <26 | 407.03 | 6384 |
| W288 | 1 | Control | Female | <26 | 228.85 | 6424 |
| L539 | 1 | Control | Female | <26 | 138.72 | 7445 |
| C500 | 1 | Control | Male | <26 | 192.98 | 8345 |
| L283 | 1 | Control | Male | <26 | 262.29 | 8674 |
| S380 | 1 | Control | Female | <26 | 721.09 | 9275 |
| N249 | 1 | Control | Female | <26 | 271.73 | 9588 |
| I688 | 1 | Control | Female | <26 | 893 | 9717 |
| N250 | 1 | Control | Female | <26 | 59 | 10528 |
| B364 | 1 | Control | Male | <26 | 213.89 | 11200 |
| F290 | 1 | Control | Female | <26 | 730.77 | 11222 |
| X728 | 1 | Control | Female | <26 | 212.88 | 11640 |
| W272 | 1 | Control | Female | <26 | 284.54 | 12335 |
| D685 | 1 | Control | Female | <26 | 151.38 | 12597 |
| R876 | 1 | Control | Female | <26 | 97.34 | 12658 |
| N000 | 1 | Control | Female | <26 | 934 | 13588 |
| T676 | 1 | Control | Female | <26 | 447.58 | 13817 |
| J110 | 1 | Control | Male | <26 | 45.82 | 16918 |
| B654 | 1 | Control | Female | <26 | 2307 | 19789 |
| G165 | 1 | Control | Female | <26 | 326.38 | 19866 |
| S830 | 1 | Control | Female | <26 | 224.92 | 21388 |
| K175 | 1 | Control | Female | <26 | 550.39 | 21860 |
| N563 | 1 | Control | Female | <26 | 919.55 | 22663 |
| A135 | 1 | Control | Male | <26 | 181.08 | 23611 |
| S492 | 1 | Control | Female | <26 | 408.76 | 24362 |
| Y827 | 1 | Control | Male | <26 | 519.77 | 24545 |
| Q591 | 1 | Control | Female | <26 | 944.74 | 25228 |
| F786 | 1 | Control | Male | <26 | 472.3 | 30597 |
| J419 | 1 | Control | Female | <26 | 293.91 | 32302 |
| O818 | 1 | Control | Male | <26 | 660.27 | 37618 |
| B208 | 1 | Control | Female | <26 | 180.9 | 39641 |
| R458 | 1 | Control | Female | <26 | 749 | 43072 |
| F711 | 1 | Control | Female | <26 | 559.9 | 48312 |
| C646 | 1 | Control | Female | <26 | 835.12 | 54586 |
| W889 | 1 | Control | Female | <26 | 298.05 | 90840 |
| D431 | 1 | Control | Male | <26 | 201.04 | 118530 |
| Q981 | 1 | Control | Female | >26 | 800 | 261150 |
| Z676 | 1 | Control | Female | <26 | 3.1 |  |
| M426 | 1 | Control | Male | <26 | 121.77 |  |
| E215 | 1 | Control | Male | <26 | 529.66 |  |
| K509 | 1 | Control | Female | <26 | 3708 |  |
| E435 | 1 | Prophylactic | Female | <26 | 15.54 | 321 |
| E418 | 1 | Prophylactic | Female | <26 | 5.35 | 340 |
| M958 | 1 | Prophylactic | Male | <26 | 36.71 | 393 |
| I951 | 1 | Prophylactic | Male | <26 | 51.54 | 407 |
| A335 | 1 | Prophylactic | Male | <26 | 84.29 | 419 |
| D321 | 1 | Prophylactic | Female | <26 | 66.56 | 437 |
| J179 | 1 | Prophylactic | Male | <26 | 46.49 | 459 |
| U469 | 1 | Prophylactic | Male | <26 | 3.1 | 467 |
| A121 | 1 | Prophylactic | Female | <26 | 119.05 | 475 |
| X556 | 1 | Prophylactic | Male | <26 | 8.29 | 544 |
| U458 | 1 | Prophylactic | Female | <26 | 103.09 | 570 |
| K100 | 1 | Prophylactic | Female | <26 | 582.05 | 595 |
| J470 | 1 | Prophylactic | Female | <26 | 44.88 | 637 |
| V904 | 1 | Prophylactic | Female | <26 | 85.66 | 895 |
| T707 | 1 | Prophylactic | Female | <26 | 373.9 | 917 |
| S191 | 1 | Prophylactic | Female | <26 | 130.48 | 924 |
| G535 | 1 | Prophylactic | Male | <26 | 111.38 | 1129 |
| B152 | 1 | Prophylactic | Female | <26 | 173.18 | 1183 |
| T893 | 1 | Prophylactic | Male | <26 | 24.83 | 1254 |
| A739 | 1 | Prophylactic | Female | <26 | 169.35 | 1284 |
| Q474 | 1 | Prophylactic | Male | <26 | 329.79 | 1290 |
| B968 | 1 | Prophylactic | Female | <26 | 170.82 | 1414 |
| A949 | 1 | Prophylactic | Female | <26 | 64.48 | 1470 |
| B621 | 1 | Prophylactic | Male | <26 | 75.08 | 1632 |
| N838 | 1 | Prophylactic | Female | <26 | 109.45 | 1723 |
| C997 | 1 | Prophylactic | Female | <26 | 164.89 | 1817 |
| X519 | 1 | Prophylactic | Female | <26 | 113.35 | 2064 |
| Z793 | 1 | Prophylactic | Female | <26 | 440.83 | 2409 |
| A637 | 1 | Prophylactic | Male | <26 | 62.25 | 3208 |
| M287 | 1 | Prophylactic | Female | <26 | 150.61 | 3479 |
| H581 | 1 | Prophylactic | Male | <26 | 547.89 | 3589 |
| Y185 | 1 | Prophylactic | Female | <26 | 147.96 | 3702 |
| O690 | 1 | Prophylactic | Female | <26 | 185.16 | 3742 |
| Z731 | 1 | Prophylactic | Female | <26 | 177.8 | 4148 |
| L575 | 1 | Prophylactic | Male | <26 | 16.19 | 5147 |
| N227 | 1 | Prophylactic | Female | <26 | 257.63 | 5435 |
| W858 | 1 | Prophylactic | Male | <26 | 48.93 | 5622 |
| L400 | 1 | Prophylactic | Female | <26 | 125.35 | 6166 |
| X218 | 1 | Prophylactic | Female | <26 | 96.94 | 6682 |
| M238 | 1 | Prophylactic | Female | <26 | 775 | 6693 |
| D229 | 1 | Prophylactic | Female | <26 | 229.8 | 7360 |
| I338 | 1 | Prophylactic | Female | <26 | 363.71 | 7402 |
| M609 | 1 | Prophylactic | Female | <26 | 561.3 | 7970 |
| J141 | 1 | Prophylactic | Female | <26 | 96.32 | 8103 |
| H504 | 1 | Prophylactic | Female | <26 | 1562 | 8507 |
| R715 | 1 | Prophylactic | Male | <26 | 300.81 | 8567 |
| D447 | 1 | Prophylactic | Female | <26 | 280.95 | 8992 |
| H782 | 1 | Prophylactic | Female | <26 | 185.26 | 9846 |
| V512 | 1 | Prophylactic | Female | <26 | 912.45 | 10206 |
| F272 | 1 | Prophylactic | Female | <26 | 797.68 | 12234 |
| E551 | 1 | Prophylactic | Female | <26 | 972 | 13028 |
| L223 | 1 | Prophylactic | Male | <26 | 135.85 | 13716 |
| S497 | 1 | Prophylactic | Female | <26 | 727.47 | 14067 |
| Z231 | 1 | Prophylactic | Female | <26 | 281.64 | 14268 |
| K510 | 1 | Prophylactic | Female | <26 | 227.3 | 16151 |
| P560 | 1 | Prophylactic | Female | <26 | 721 | 16574 |
| N259 | 1 | Prophylactic | Female | <26 | 831 | 19160 |
| I840 | 1 | Prophylactic | Male | <26 | 441.87 | 19219 |
| T607 | 1 | Prophylactic | Female | <26 | 502.71 | 19309 |
| J928 | 1 | Prophylactic | Male | <26 | 74.07 | 19499 |
| V875 | 1 | Prophylactic | Female | <26 | 858 | 19924 |
| G100 | 1 | Prophylactic | Male | <26 | 22.58 | 22596 |
| U900 | 1 | Prophylactic | Female | <26 | 124.69 | 26249 |
| U149 | 1 | Prophylactic | Female | <26 | 665.21 | 26521 |
| Q907 | 1 | Prophylactic | Female | <26 | 240.15 | 26923 |
| W385 | 1 | Prophylactic | Male | <26 | 77.85 | 27271 |
| V844 | 1 | Prophylactic | Male | <26 | 651.5 | 31016 |
| G941 | 1 | Prophylactic | Male | <26 | 80.25 | 33093 |
| T214 | 1 | Prophylactic | Female | <26 | 696.11 | 37606 |
| E111 | 1 | Prophylactic | Male | <26 | 184.63 | 49306 |
| X171 | 1 | Prophylactic | Male | <26 | 638 | 60787 |
| C641 | 1 | Prophylactic | Female | <26 | 21.37 |  |
| K565 | 1 | Prophylactic | Female | <26 | 63.34 |  |
| Z000 | 1 | Prophylactic | Male | <26 | 70.26 |  |
| R848 | 1 | Prophylactic | Male | <26 | 163.89 |  |
| V755 | 1 | Prophylactic | Female | <26 | 196.15 |  |
| H296 | 1 | Prophylactic | Female | <26 | 432.87 |  |
| H113 | 2 | Control | Male | <26 | 4.33 | 16.65 |
| W861 | 2 | Control | Male | <26 | 3.68 | 30.8 |
| Q309 | 2 | Control | Male | <26 | 5.03 | 51.18 |
| N452 | 2 | Control | Female | <26 | 23.6 | 80.42 |
| H988 | 2 | Control | Female | <26 | 9.83 | 190.36 |
| D006 | 2 | Control | Female | <26 | 17.5 | 392 |
| M839 | 2 | Control | Female | <26 | 38 | 421.81 |
| J162 | 2 | Control | Female | <26 | 17.84 | 601 |
| H842 | 2 | Control | Female | <26 | 1108 | 1108 |
| F787 | 2 | Control | Female | <26 | 90.3 | 1189 |
| J798 | 2 | Control | Male | >26 | 52.24 | 1208 |
| R544 | 2 | Control | Male | <26 | 97.9 | 1308 |
| U194 | 2 | Control | Female | >26 | 5.13 | 1421 |
| K407 | 2 | Control | Female | <26 | 35.26 | 1604 |
| J660 | 2 | Control | Female | <26 | 132.1 | 1670 |
| V127 | 2 | Control | Female | >26 | 16.86 | 2250 |
| P178 | 2 | Control | Female | <26 | 272.54 | 2415 |
| K341 | 2 | Control | Female | <26 | 50.78 | 2701 |
| Q923 | 2 | Control | Female | <26 | 375.06 | 2934 |
| A244 | 2 | Control | Female | <26 | 102.52 | 3421 |
| J346 | 2 | Control | Female | <26 | 190.03 | 3448 |
| S315 | 2 | Control | Female | <26 | 672.08 | 4052 |
| A243 | 2 | Control | Female | <26 | 427.57 | 4316 |
| W751 | 2 | Control | Female | <26 | 299.42 | 4964 |
| M120 | 2 | Control | Female | <26 | 1388 | 6084 |
| V060 | 2 | Control | Female | <26 | 446.81 | 6371 |
| A289 | 2 | Control | Female | <26 | 349.59 | 6374 |
| P003 | 2 | Control | Female | <26 | 48.2 | 7251 |
| O487 | 2 | Control | Female | <26 | 1318 | 9100 |
| S341 | 2 | Control | Female | <26 | 468.18 | 9199 |
| O852 | 2 | Control | Male | <26 | 166.75 | 9451 |
| J258 | 2 | Control | Female | <26 | 423.66 | 9644 |
| B641 | 2 | Control | Female | <26 | 200.08 | 10315 |
| I181 | 2 | Control | Female | <26 | 193.24 | 11326 |
| S366 | 2 | Control | Female | <26 | 326.38 | 11854 |
| M865 | 2 | Control | Female | <26 | 732.73 | 12135 |
| N118 | 2 | Control | Male | <26 | 36.61 | 12371 |
| I523 | 2 | Control | Female | <26 | 551.66 | 12760 |
| A627 | 2 | Control | Male | <26 | 195.84 | 12904 |
| I386 | 2 | Control | Male | <26 | 806.39 | 13264 |
| C118 | 2 | Control | Female | <26 | 77.4 | 13514 |
| S182 | 2 | Control | Female | <26 | 531.73 | 15688 |
| W035 | 2 | Control | Male | <26 | 65.93 | 16526 |
| X144 | 2 | Control | Female | <26 | 245.94 | 19398 |
| T486 | 2 | Control | Female | <26 | 61.39 | 26200 |
| G355 | 2 | Control | Female | >26 | 4847 | 27898 |
| E395 | 2 | Control | Male | <26 | 884.91 | 28980 |
| I407 | 2 | Control | Male | <26 | 371.35 | 29482 |
| N865 | 2 | Control | Female | <26 | 118.89 | 30052 |
| R379 | 2 | Control | Female | <26 | 859.7 | 30616 |
| B456 | 2 | Control | Female | <26 | 369.04 | 30617 |
| G288 | 2 | Control | Female | <26 | 362.63 | 32133 |
| X333 | 2 | Control | Female | <26 | 552.21 | 32631 |
| T652 | 2 | Control | Male | <26 | 235.17 | 53975 |
| K706 | 2 | Control | Female | <26 | 645.18 | 67596 |
| R110 | 2 | Control | Male | <26 | 840.66 | 69207 |
| S257 | 2 | Control | Male | <26 | 254.62 | 73114 |
| D048 | 2 | Control | Male | <26 | 1328 | 78075 |
| S579 | 2 | Control | Female | <26 | 613.12 | 90531 |
| A049 | 2 | Control | Male | <26 | 245.02 |  |
| E169 | 2 | Control | Female | <26 | 182610 |  |
| J362 | 2 | Control | Female | <26 | 347.41 |  |
| N046 | 2 | Control | Female | <26 | 205.54 |  |
| U327 | 2 | Control | Male | <26 | 378.96 |  |
| X162 | 2 | Control | Male | <26 | 3245 |  |
| V759 | 2 | Prophylactic | Female | >26 | 8.04 | 31.96 |
| Y485 | 2 | Prophylactic | Male | <26 | 3.8 | 37.25 |
| I342 | 2 | Prophylactic | Male | <26 | 9.41 | 139.09 |
| K585 | 2 | Prophylactic | Male | <26 | 3.1 | 189.08 |
| J440 | 2 | Prophylactic | Female | >26 | 51.08 | 326 |
| R813 | 2 | Prophylactic | Female | >26 | 31.15 | 329 |
| V237 | 2 | Prophylactic | Female | <26 | 25.32 | 403 |
| A233 | 2 | Prophylactic | Female | <26 | 12.13 | 438 |
| O706 | 2 | Prophylactic | Male | <26 | 53.11 | 503 |
| G520 | 2 | Prophylactic | Male | >26 | 52.07 | 546 |
| H098 | 2 | Prophylactic | Female | <26 | 65.79 | 739 |
| P323 | 2 | Prophylactic | Female | <26 | 346.9 | 870 |
| D956 | 2 | Prophylactic | Male | <26 | 15.94 | 930 |
| S950 | 2 | Prophylactic | Male | <26 | 80.17 | 1043 |
| Q922 | 2 | Prophylactic | Female | <26 | 283.11 | 1375 |
| U637 | 2 | Prophylactic | Female | <26 | 215.9 | 1390 |
| N134 | 2 | Prophylactic | Female | <26 | 99.75 | 1795 |
| C150 | 2 | Prophylactic | Female | <26 | 322.79 | 2377 |
| B581 | 2 | Prophylactic | Female | <26 | 406.74 | 2439 |
| V548 | 2 | Prophylactic | Male | <26 | 141.7 | 3183 |
| D143 | 2 | Prophylactic | Male | <26 | 73.36 | 3449 |
| P241 | 2 | Prophylactic | Female | <26 | 893 | 5890 |
| Z591 | 2 | Prophylactic | Female | <26 | 670.17 | 5966 |
| X219 | 2 | Prophylactic | Male | <26 | 162.87 | 6270 |
| A815 | 2 | Prophylactic | Female | <26 | 113.73 | 6426 |
| D284 | 2 | Prophylactic | Female | <26 | 6109 | 8740 |
| R617 | 2 | Prophylactic | Female | <26 | 228.83 | 9297 |
| M267 | 2 | Prophylactic | Female | >26 | 2429 | 9554 |
| M051 | 2 | Prophylactic | Male | <26 | 726.5 | 9603 |
| R298 | 2 | Prophylactic | Female | <26 | 668.84 | 9608 |
| Q114 | 2 | Prophylactic | Female | <26 | 302.86 | 10542 |
| Y938 | 2 | Prophylactic | Female | <26 | 634.53 | 11281 |
| U412 | 2 | Prophylactic | Male | <26 | 134.86 | 12304 |
| I864 | 2 | Prophylactic | Female | <26 | 110.43 | 12970 |
| O293 | 2 | Prophylactic | Male | <26 | 30.38 | 13498 |
| I607 | 2 | Prophylactic | Female | <26 | 8817 | 19804 |
| C532 | 2 | Prophylactic | Female | <26 | 828 | 19870 |
| K496 | 2 | Prophylactic | Male | >26 | 42.32 | 20584 |
| Z638 | 2 | Prophylactic | Female | <26 | 301.69 | 22826 |
| Q669 | 2 | Prophylactic | Female | <26 | 1449 | 23041 |
| E833 | 2 | Prophylactic | Female | >26 | 16447 | 24710 |
| Z576 | 2 | Prophylactic | Female | <26 | 107.07 | 28556 |
| Z982 | 2 | Prophylactic | Female | <26 | 467.57 | 28908 |
| E423 | 2 | Prophylactic | Female | <26 | 21305 | 30489 |
| B104 | 2 | Prophylactic | Female | <26 | 791.2 | 31672 |
| X874 | 2 | Prophylactic | Female | <26 | 833.64 | 35343 |
| X351 | 2 | Prophylactic | Female | <26 | 1215 | 78066 |
| J769 | 2 | Prophylactic | Male | <26 | 2838 | 81655 |
| F973 | 2 | Prophylactic | Female | <26 | 1375 | 198900 |
| B229 | 2 | Prophylactic | Male | <26 | 88893 |  |
| F569 | 2 | Prophylactic | Male | <26 | 86.7 |  |
| G017 | 2 | Prophylactic | Male | <26 | 36628 |  |
| H365 | 2 | Prophylactic | Male | <26 | 20.58 |  |
| L263 | 2 | Prophylactic | Male | <26 | 73.28 |  |
| L978 | 2 | Prophylactic | Male | <26 | 882.15 |  |
| P349 | 2 | Prophylactic | Female | <26 | 26.36 |  |
| W530 | 2 | Therapeutic | Female | <26 | 3.1 | 13.81 |
| K578 | 2 | Therapeutic | Male | <26 | 4.72 | 22.64 |
| O743 | 2 | Therapeutic | Female | >26 | 3.1 | 29.24 |
| Z384 | 2 | Therapeutic | Female | <26 | 4.51 | 35.01 |
| Y184 | 2 | Therapeutic | Male | <26 | 10.72 | 47.92 |
| W446 | 2 | Therapeutic | Female | <26 | 35.38 | 93.54 |
| N913 | 2 | Therapeutic | Female | <26 | 3.1 | 117.14 |
| F389 | 2 | Therapeutic | Male | <26 | 4.15 | 131.84 |
| B201 | 2 | Therapeutic | Male | <26 | 23.38 | 146.06 |
| S072 | 2 | Therapeutic | Male | <26 | 55.72 | 156.77 |
| E557 | 2 | Therapeutic | Male | <26 | 3.1 | 201.26 |
| K802 | 2 | Therapeutic | Male | <26 | 19.88 | 306.7 |
| A750 | 2 | Therapeutic | Female | <26 | 56.7 | 343 |
| O380 | 2 | Therapeutic | Male | <26 | 83.8 | 456 |
| Z720 | 2 | Therapeutic | Female | <26 | 62.51 | 706 |
| I013 | 2 | Therapeutic | Male | <26 | 45.63 | 788 |
| T109 | 2 | Therapeutic | Female | >26 | 16.6 | 811 |
| H378 | 2 | Therapeutic | Female | <26 | 212.85 | 1097 |
| H848 | 2 | Therapeutic | Female | <26 | 108.82 | 1108 |
| O579 | 2 | Therapeutic | Male | <26 | 280.87 | 1248 |
| M875 | 2 | Therapeutic | Female | <26 | 75.91 | 1507 |
| Z274 | 2 | Therapeutic | Male | <26 | 98.96 | 1530 |
| L760 | 2 | Therapeutic | Female | <26 | 89.81 | 1663 |
| J966 | 2 | Therapeutic | Female | <26 | 140.72 | 1691 |
| F497 | 2 | Therapeutic | Male | <26 | 200.08 | 1878 |
| F595 | 2 | Therapeutic | Female | <26 | 205.7 | 1966 |
| E967 | 2 | Therapeutic | Male | <26 | 123.09 | 2074 |
| P129 | 2 | Therapeutic | Male | <26 | 158.08 | 2112 |
| J756 | 2 | Therapeutic | Female | <26 | 83.3 | 2217 |
| U650 | 2 | Therapeutic | Female | <26 | 525.48 | 2358 |
| N704 | 2 | Therapeutic | Male | <26 | 156.49 | 2400 |
| V935 | 2 | Therapeutic | Female | <26 | 31.4 | 2401 |
| C222 | 2 | Therapeutic | Female | <26 | 406.36 | 2514 |
| Y957 | 2 | Therapeutic | Female | <26 | 80.73 | 2608 |
| H796 | 2 | Therapeutic | Female | <26 | 97.1 | 2704 |
| G278 | 2 | Therapeutic | Male | <26 | 7.25 | 2740 |
| X483 | 2 | Therapeutic | Male | <26 | 40.64 | 2913 |
| A999 | 2 | Therapeutic | Male | <26 | 326.49 | 3083 |
| Y713 | 2 | Therapeutic | Female | <26 | 104.18 | 3281 |
| P036 | 2 | Therapeutic | Female | <26 | 775.79 | 3399 |
| M721 | 2 | Therapeutic | Male | <26 | 3.1 | 3438 |
| K673 | 2 | Therapeutic | Female | <26 | 174.61 | 3569 |
| O102 | 2 | Therapeutic | Female | <26 | 133.05 | 3705 |
| F063 | 2 | Therapeutic | Female | <26 | 379.1 | 3731 |
| O850 | 2 | Therapeutic | Male | <26 | 114.24 | 3767 |
| R321 | 2 | Therapeutic | Female | <26 | 110.91 | 3835 |
| L077 | 2 | Therapeutic | Female | <26 | 266.15 | 4270 |
| C099 | 2 | Therapeutic | Female | <26 | 178.06 | 4423 |
| Q716 | 2 | Therapeutic | Female | <26 | 80.29 | 4897 |
| B062 | 2 | Therapeutic | Male | <26 | 419.74 | 5343 |
| O092 | 2 | Therapeutic | Female | <26 | 474.12 | 5354 |
| H918 | 2 | Therapeutic | Female | <26 | 299.31 | 6157 |
| E689 | 2 | Therapeutic | Male | <26 | 114.17 | 6864 |
| N457 | 2 | Therapeutic | Female | <26 | 45.79 | 7392 |
| W394 | 2 | Therapeutic | Female | <26 | 449.89 | 7938 |
| A358 | 2 | Therapeutic | Male | <26 | 153.93 | 7941 |
| D411 | 2 | Therapeutic | Male | <26 | 450.77 | 7950 |
| Y589 | 2 | Therapeutic | Female | <26 | 293.49 | 8188 |
| A387 | 2 | Therapeutic | Female | <26 | 339.27 | 8263 |
| H699 | 2 | Therapeutic | Female | <26 | 355.82 | 8540 |
| Z681 | 2 | Therapeutic | Female | <26 | 290.12 | 8862 |
| F239 | 2 | Therapeutic | Female | <26 | 248.26 | 8952 |
| L882 | 2 | Therapeutic | Female | <26 | 522.74 | 9254 |
| T578 | 2 | Therapeutic | Female | <26 | 374.44 | 10146 |
| R438 | 2 | Therapeutic | Female | <26 | 430.07 | 10214 |
| O304 | 2 | Therapeutic | Female | <26 | 320.69 | 11475 |
| W513 | 2 | Therapeutic | Female | <26 | 471.68 | 12268 |
| F970 | 2 | Therapeutic | Female | <26 | 510.77 | 12313 |
| Q553 | 2 | Therapeutic | Female | <26 | 789.35 | 14287 |
| W509 | 2 | Therapeutic | Male | >26 | 156.19 | 14447 |
| D714 | 2 | Therapeutic | Male | <26 | 3485 | 14629 |
| L035 | 2 | Therapeutic | Female | <26 | 605.74 | 14846 |
| C895 | 2 | Therapeutic | Female | <26 | 421.25 | 15781 |
| I478 | 2 | Therapeutic | Female | <26 | 44.16 | 16481 |
| F843 | 2 | Therapeutic | Female | <26 | 134.71 | 16706 |
| B557 | 2 | Therapeutic | Female | <26 | 29.9 | 17100 |
| F040 | 2 | Therapeutic | Female | <26 | 36.79 | 17945 |
| J892 | 2 | Therapeutic | Female | <26 | 145.26 | 19075 |
| Y481 | 2 | Therapeutic | Female | <26 | 25.52 | 19407 |
| T901 | 2 | Therapeutic | Female | <26 | 821.82 | 20732 |
| Y039 | 2 | Therapeutic | Female | <26 | 886 | 21906 |
| P520 | 2 | Therapeutic | Female | <26 | 539.87 | 22502 |
| U786 | 2 | Therapeutic | Female | <26 | 528.12 | 25075 |
| D610 | 2 | Therapeutic | Male | <26 | 293.56 | 25799 |
| M654 | 2 | Therapeutic | Female | <26 | 157.69 | 25877 |
| Y737 | 2 | Therapeutic | Female | >26 | 544.47 | 31279 |
| Q702 | 2 | Therapeutic | Female | <26 | 108.1 | 31600 |
| L163 | 2 | Therapeutic | Female | <26 | 949.06 | 32664 |
| R269 | 2 | Therapeutic | Female | <26 | 575.91 | 34067 |
| L106 | 2 | Therapeutic | Female | <26 | 1097 | 34628 |
| Z936 | 2 | Therapeutic | Female | <26 | 945.55 | 36320 |
| M778 | 2 | Therapeutic | Male | <26 | 427.46 | 36978 |
| L869 | 2 | Therapeutic | Female | <26 | 5730 | 37633 |
| O819 | 2 | Therapeutic | Male | <26 | 229.94 | 39124 |
| U705 | 2 | Therapeutic | Female | <26 | 36509 | 45556 |
| L666 | 2 | Therapeutic | Male | <26 | 39428 | 48039 |
| F407 | 2 | Therapeutic | Female | <26 | 417.66 | 48336 |
| U544 | 2 | Therapeutic | Male | <26 | 31892 | 50795 |
| Y587 | 2 | Therapeutic | Male | <26 | 99.6 | 53319 |
| N667 | 2 | Therapeutic | Male | <26 | 170.03 | 54008 |
| H302 | 2 | Therapeutic | Male | <26 | 314.16 | 61962 |
| E248 | 2 | Therapeutic | Female | <26 | 373.13 | 62966 |
| D362 | 2 | Therapeutic | Female | <26 | 851.51 | 72374 |
| D616 | 2 | Therapeutic | Female | <26 | 254.04 | 75695 |
| P427 | 2 | Therapeutic | Male | <26 | 63703 | 76166 |
| M706 | 2 | Therapeutic | Female | <26 | 2218 | 86225 |
| V485 | 2 | Therapeutic | Male | <26 | 880.18 | 178600 |
| C414 | 2 | Therapeutic | Female | <26 | 18.54 |  |
| K618 | 2 | Therapeutic | Female | <26 | 171.31 |  |
| M644 | 2 | Therapeutic | Female | <26 | 537.12 |  |
| N085 | 2 | Therapeutic | Male | <26 | 254.51 |  |
| N380 | 2 | Therapeutic | Female | <26 | 281.78 |  |
| T052 | 2 | Therapeutic | Female | <26 | 441.41 |  |
| T443 | 2 | Therapeutic | Male | <26 | 81.9 |  |
| T621 | 2 | Therapeutic | Male | <26 | 269.79 |  |
| * Anti-HBs levels measured directly before (1) and one month after (2) the second booster vaccination. | | | | | | |
