# Supplementary material for: Effects of Prophylactic and Therapeutic Paracetamol Treatment during Vaccination on Hepatitis B Antibody Levels in Adults: Two Open-Label, Randomized Controlled Trials
Source: PLoS One. 2014 Jun 4;9(6):e98175. doi: 10.1371/journal.pone.0098175 (PMC4045752; doi:10.1371/journal.pone.0098175)
Supplement: Table S2 — Geometric concentrations (GMC) with 95% conficence intervals of the treatment groups. (DOCX) [file pone.0098175.s002.docx]

**Table S2. Geometric concentrations (GMC) with 95% conficence intervals of the treatment groups**

| **Phase** | **Data** | **Control (IU/L)** | **Prophylactic (IU/L)** | **Therapeutic (IU/L)** |
| --- | --- | --- | --- | --- |
| 1 | Raw | 5846 (3784-9022) | 4116 (2894-5854) | - |
| 1 | Corrected | 6166 (3991-9528) | 4342 (3053-6175) | - |
|  |  |  |  |  |
| 2 | Raw | 5387 (3231-8983) | 4160 (2345-7379) | 4984 (3398-7312) |
| 2 | Corrected | 5358 (3214-8934) | 4138 (2333-7339) | 4958 (3380-7272) |
|  |  |  |  |  |
| 1 + 2 | Raw | 5623 (4048-7812) | 4134 (3037-5627) | 4984 (3398-7312) |
| 1 + 2 | Corrected | 5768 (4151-8014) | 4257 (3128-5795) | 4958 (3380-7272) |
