# Supplementary material for: Effects of Prophylactic and Therapeutic Paracetamol Treatment during Vaccination on Hepatitis B Antibody Levels in Adults: Two Open-Label, Randomized Controlled Trials
Source: PLoS One. 2014 Jun 4;9(6):e98175. doi: 10.1371/journal.pone.0098175 (PMC4045752; doi:10.1371/journal.pone.0098175)
Supplement: Protocol S1 — Trial Protocol. (DOC) [file pone.0098175.s004.doc]

**RESEARCH PROTOCOL**

**Paracetamol and Hepatitis B Vaccination**

**(juli 2012)**

**PROTOCOL TITLE** ‘Effects of paracetamol on the immune response after Hepatitis B vaccination.’

| **Protocol ID** | HepB/APAP/2011 |
| --- | --- |
| **Short title** | Paracetamol and Hepatitis B Vaccination |
| **EudraCT number** | 2011-000923-33 |
| **Version** | 04 |
| **Date** | 09-07-2012 |
| **Coordinating investigator/project leader** | Dr. R. Janssen  Centre for Health Protection (GZB), National Institute for Public Health and the Environment (RIVM), The Netherlands |
| **Investigator(s)** | A.M.C.M. Doedée, MSc  Centre for Health Protection (GBO), National Institute for Public Health and the Environment (RIVM), The Netherlands  Dr. G.J. Boland  Medical Microbiology and Virology, University Medical Centre Utrecht (UMCU), The Netherlands |
| **Sponsor (in Dutch: verrichter/opdrachtgever)** | National Institute of Health and the Environment (RIVM), The Netherlands |
| **Independent expert (s)** | Dr. T. Wolfs  Kinderarts WKZ/UMC Utrecht |
| **Laboratory sites** | RIVM: Centre for Health Protection (GZB)  UMCU: Medical Microbiology and Virology |
| **Pharmacy** | *Paracetamol: Pharmacy Bilthoven* |
|  | *Hepatitis B vaccine: Pharmacy UMC Utrecht* |

**PROTOCOL SIGNATURE SHEET**

| **Name** | **Signature** | **Date** |
| --- | --- | --- |
| Sponsor or legal representative:  *National Institute for Public Health and the Environment, Centre for Infectious Diseases;*  *Prof. Dr. R.A. Coutinho*  Head of Department (Centre for Public Health);  *Dr. Annemieke de Vries* |  |  |
| Coordinating Investigator/Project leader; Dr. R. Janssen  Investigator; Anne Doedée, MSc  Dr. G.J. Boland |  |  |

**TABLE OF CONTENTS**

1. INTRODUCTION AND RATIONALE [10](#__RefHeading___Toc372715541)

2. OBJECTIVES [12](#__RefHeading___Toc372715542)

2.1 Primary Objective [12](#__RefHeading___Toc372715543)

2.2 Secondary Objective [12](#__RefHeading___Toc372715544)

3. STUDY DESIGN [13](#__RefHeading___Toc372715545)

4. STUDY POPULATION [14](#__RefHeading___Toc372715546)

4.1 Population (base) [14](#__RefHeading___Toc372715547)

4.2 Inclusion criteria [14](#__RefHeading___Toc372715548)

4.3 Exclusion criteria [14](#__RefHeading___Toc372715549)

4.4 Sample size calculation [15](#__RefHeading___Toc372715550)

5. TREATMENT OF SUBJECTS [19](#__RefHeading___Toc372715551)

5.1 Investigational product/treatment [19](#__RefHeading___Toc372715552)

5.2 Use of co-intervention [19](#__RefHeading___Toc372715553)

5.3 Escape medication [20](#__RefHeading___Toc372715554)

6. INVESTIGATIONAL MEDICINAL PRODUCT [20](#__RefHeading___Toc372715555)

7. METHODS [21](#__RefHeading___Toc372715556)

7.1 Study parameters/endpoints [21](#__RefHeading___Toc372715557)

7.1.1 Main study parameter/endpoint [21](#__RefHeading___Toc372715558)

7.1.2 Secondary study parameters/endpoints [21](#__RefHeading___Toc372715559)

7.2 Randomisation, blinding and treatment allocation [21](#__RefHeading___Toc372715560)

7.3 Study procedures [22](#__RefHeading___Toc372715561)

7.4 Withdrawal of individual subjects [23](#__RefHeading___Toc372715562)

7.5 Replacement of individual subjects after withdrawal [23](#__RefHeading___Toc372715563)

7.6 Follow-up of subjects withdrawn from treatment no follow-up [23](#__RefHeading___Toc372715564)

7.7 Premature termination of the study [23](#__RefHeading___Toc372715565)

8. SAFETY REPORTING [24](#__RefHeading___Toc372715566)

8.1 Section 10 WMO event [24](#__RefHeading___Toc372715567)

8.2 AEs, SAEs and SUSARs [24](#__RefHeading___Toc372715568)

8.2.1 Adverse events (AEs) [24](#__RefHeading___Toc372715569)

8.2.2 Serious adverse events (SAEs) [24](#__RefHeading___Toc372715570)

8.2.3 Suspected unexpected serious adverse reactions (SUSARs) [25](#__RefHeading___Toc372715571)

8.3 Annual safety report [26](#__RefHeading___Toc372715572)

8.4 Follow-up of adverse events [26](#__RefHeading___Toc372715573)

8.5 Data Safety Monitoring Board (DSMB) [26](#__RefHeading___Toc372715574)

9. STATISTICAL ANALYSIS [28](#__RefHeading___Toc372715575)

9.1 Descriptive statistics [28](#__RefHeading___Toc372715576)

9.2 Univariate analysis [28](#__RefHeading___Toc372715577)

10. ETHICAL CONSIDERATIONS [30](#__RefHeading___Toc372715578)

10.1 Regulation statement [30](#__RefHeading___Toc372715579)

10.2 Recruitment and consent [30](#__RefHeading___Toc372715580)

10.3 Benefits and risks assessment, group relatedness [31](#__RefHeading___Toc372715581)

10.4 Compensation for injury [31](#__RefHeading___Toc372715582)

10.5 Incentives [31](#__RefHeading___Toc372715583)

11. ADMINISTRATIVE ASPECTS, MONITORING AND PUBLICATION [31](#__RefHeading___Toc372715584)

11.1 Handling and storage of data and documents [31](#__RefHeading___Toc372715585)

11.2 Amendments [32](#__RefHeading___Toc372715586)

11.3 Annual progress report [32](#__RefHeading___Toc372715587)

11.4 End of study report [32](#__RefHeading___Toc372715588)

11.5 Public disclosure and publication policy [33](#__RefHeading___Toc372715589)

12. REFERENCES [33](#__RefHeading___Toc372715590)

**LIST OF ABBREVIATIONS AND RELEVANT DEFINITIONS**

| **ABR** | **ABR form, General Assessment and Registration form, is the application form that is required for submission to the accredited Ethics Committee (In Dutch, ABR = Algemene Beoordeling en Registratie)** |
| --- | --- |
| **AE** | **Adverse Event** |
| **AR** | **Adverse Reaction** |
| **CA** | **Competent Authority** |
| **CCMO** | **Central Committee on Research Involving Human Subjects; in Dutch: Centrale Commissie Mensgebonden Onderzoek** |
| **CV** | **Curriculum Vitae** |
| **DSMB** | **Data Safety Monitoring Board** |
| **EU** | **European Union** |
| **EudraCT** | **European drug regulatory affairs Clinical Trials** |
| **GCP** | **Good Clinical Practice** |
| **IB** | **Investigator’s Brochure** |
| **IC** | **Informed Consent** |
| **IMP** | **Investigational Medicinal Product** |
| **IMPD** | **Investigational Medicinal Product Dossier** |
| **METC** | **Medical research ethics committee (MREC); in Dutch: medisch ethische toetsing commissie (METC)** |
| **NIP** | **National Immunization Program** |
| **(S)AE** | **(Serious) Adverse Event** |
| **SPC** | **Summary of Product Characteristics** |
| **Sponsor** | **The sponsor is the party that commissions the organisation or performance of the research, for example a pharmaceutical**  **company, academic hospital, scientific organisation or investigator. A party that provides funding for a study but does not commission it is not regarded as the sponsor, but referred to as a subsidising party.** |
| **SUSAR** | **Suspected Unexpected Serious Adverse Reaction** |
| **Wbp** | **Personal Data Protection Act (in Dutch: Wet Bescherming Persoonsgevens)** |
| **WMO** | **Medical Research Involving Human Subjects Act (in Dutch: Wet Medisch-wetenschappelijk Onderzoek met Mensen** |

**SUMMARY**

**Rationale:** Paracetamol (acetaminophen) is a medicine that is widely used in children and adults to relieve pain and reduce fever. In many European countries, paracetamol is used after vaccination. In the Netherlands, the advice is to be reserved with the use of paracetamol during vaccination and only children who experienced fever or persistent screaming after vaccination are advised to use paracetamol before further vaccinations. Despite this advice to restrict paracetamol to those children with previous reactions, many parents give paracetamol to their children before or just after the vaccine administrations according to the National Immunization Program (NIP).

Paracetamol is generally regarded as safe and is commonly used as an over-the-counter drug. Paracetamol was long considered to be a drug without effects on the immune system. Recently published results suggest a negative influence of paracetamol on the vaccination response. Infants who received paracetamol directly after vaccination to prevent fever had a lower vaccination response. The effects of paracetamol on the immune system could affect the protection level of vaccination. At this moment, it is not known if paracetamol has the same effects in adults. Also, it is not known if there is an important role for timing of paracetamol in this effect on vaccination response. It is important to gain insight into the effects of paracetamol use during vaccination. Health organizations should have enough information to offer good advice about the use of paracetamol during vaccination procedures.

**Objective:** Phase 1: Investigate a possible suppressing effect of prophylactic paracetamol use in adults on the response of the hepatitis B vaccination.

Phase 2: Study the association between timing of the use of paracetamol during hepatitis vaccination and the development of the immune response in adults.

**Study design:** Two-phase study. In phase 1 we will investigate if prophylactic use of paracetamol has an effect on the immune response of adults after a hepatitis B vaccination. In phase 2 we will investigate if there is a difference between prophylactic and therapeutic use of paracetamol on the vaccination response. The first month of phase 1 and 2 of the study is interventional, after that it will turn into an observational study. It is important to realize that we only start with phase 2 if we find any relevant effects of paracetamol on the immune response after vaccination in phase 1.

**Study population:**  Healthy young health care students of 18 years or older, who are routinely vaccination with hepatitis B vaccine will be approached by the Hogeschool Utrecht for a hepatitis B vaccination.

**Intervention:** Phase 1: There is one intervention and one control group. The intervention group have to take paracetamol for 24 hours (1000 mg/8h) starting directly after the vaccination. The control group takes no paracetamol. Every participant will take paracetamol according to the intervention or control group during the first and second hepatitis B vaccinations.

Phase 2: There are two intervention groups that have to take paracetamol for 24 hours (1000mg/8h) and one control group. Timing of the paracetamol use is different: group 1 will take paracetamol at the time of vaccination (prophylactic use) and group 2 starts taking paracetamol 6 hours after vaccination (therapeutic use 1). Every participant will take paracetamol according to one of the three groups during the first and second hepatitis B vaccination.

**Primary study parameters/endpoints:** Phase 1: Antibody concentrations prior to and one month after the third vaccination (second booster vaccination) will be measured. Antibody concentrations expected to be 65% lower in participant that used paracetamol prophylactically. This expectation is based on finding in babies (Prymula et al., 2009).

Phase 2: Antibody concentrations prior to and one month after the third vaccination (second booster vaccination) will be measured. It is expected that the antibody concentration in the therapeutic paracetamol group will be in between the antibody concentration of the control and the prophylactic paracetamol group.

**Secondary study parameters/outcome of the study:** Alterations in the functionality of specific lymphocyte subpopulations in blood (one month after the third vaccination) of participants that prophylactically used paracetamol compared to the control group.

**Nature and extent of the burden and risks associated with participation, group relatedness:**

Participants who take paracetamol prophylactically or therapeutically will probably suffer less from side effects of vaccination, such as fever and pain. Participation to this study will significantly contribute to the scientific knowledge, resulting in a better advice of health organizations about the use of paracetamol during vaccination procedures. There is a possibility that some participants could have a reduced antibody concentration at the end of the study. The antibody concentration of every participant will be determined and evaluated, like is routinely done after hepatitis B vaccination. An extra booster vaccination will be offered if the antibody concentration is too low for protection against hepatitis B. This is the normal policy and is not different for students who participate in this study and those that do not. The study population exists of young health care students who are routinely vaccinated against hepatitis B. In the vaccination protocol a blood sample is routinely taken 1 month after the third vaccination. For this study, we will draw 21 mL. of extra blood at this time point. An extra blood sample (7 ml.) will be taken specifically for this study prior to the third vaccination. Antibody concentrations (prior to and one month after the third vaccination) and functionality of lymphocyte subpopulations (one month after the third vaccination) will be determined in the blood.

# INTRODUCTION AND RATIONALE

Paracetamol (acetaminophen) is an analgesic and antipyretic drug, commonly used both in infants and adults (Jackson et al., 2006). In many European countries paracetamol is used prophylactically to reduce pain and fever associated with vaccination as a routine (Manley, 2007). In the Netherlands this is also being considered, and children who have experienced fever after vaccination are advised to use paracetamol prior to further vaccinations. Despite this advice to restrict prior paracetamol to those children with previous reactions, many parents give paracetamol to their children prior to or after the NIP injections. Estimates of paracetamol use range from 9-40% prophylactically, and up to 49% prophylactically and therapeutically (David et al, 2009).

The most important toxic effect that occurs at high doses is hepatotoxicity, which is caused by glutathione depletion, oxidative stress, mitochondrial dysfunction and inflammatory responses. Indications that paracetamol has immunotoxic effects have emerged. Recently published results suggest a negative influence on vaccination response (lower antibody concentrations) in infants that were prophylactically administered paracetamol prior to vaccination to prevent fever (Prymula *et al*., 2009). These effects were only found after paracetamol treatment during the primary vaccinations and not after the boost vaccination. In a comparable study with influenza vaccination in adults, the effects were less evident (Gross et al, 1994). These data suggest that the effects of paracetamol are limited to the primary vaccination response. In the influenza study, adults are vaccinated against influenza viruses. However, adults are already exposed to influenza viruses during lifetime. Therefore the vaccination will not induce a primary vaccination response, but a reaction that is comparable with a booster vaccination. An alternative explanation could be that the effects of paracetamol are limited to the immune response in babies, because of the immature immune system.

In animal studies paracetamol suppressed several immune parameters, such as T-cell dependent antibody responses , and toxicogenomics studies revealed an influence on gene expression in lymphocytes compatible with suppression of proliferation, which leads to immunosuppression (Baken *et al*., 2008). This is in line with older studies that hinted at suppressive effects on chickenpox (Doran *et al*., 1989) and rhinovirus (Graham *et al*, 1990). In addition to these immunosuppressive effects, several epidemiological studies suggest that there is an association between the use of paracetamol in infants and adults and asthma . Asthma is an immunologically mediated disease and distortion of the immune system by paracetamol may be underlying this association.

There are a number of potential mechanisms whereby the frequent use of paracetamol might influence the immune system. Paracetamol might decrease the amount of reduced glutathione thereby impairing respiratory antioxidant defenses. Decreased glutathione levels can influence regulatory mechanisms of lymphocytes. Paracetamol may also influence COX-2 activity and the production of prostaglandin. Another potential mechanism for which there is conflicting evidence is that by suppressing fever, paracetamol might reduce the predominant TH1 cytokine storm that occurs as part of the febrile response (reviewed by Farquhar et al., 2009).

In this study, the primary objective is the possible suppressing effect of paracetamol use on the response to hepatitis B vaccination, and the effect of timing of paracetamol in this effect on vaccination response. The graph below shows three possible curves of the vaccination response after the primary (first peak) and booster (second peak) vaccination. Curve 1 represents a normal vaccination response. Curve 2 and 3 represent alternative vaccination responses, for example after prophylactic use of paracetamol during vaccination to prevent fever and pain. Curve 2 shows a delay of the normal response resulting in the same antibody concentration around 18 months of age. Curve 3 shows a decreased antibody concentration after the primary and booster vaccination resulting in a lower antibody concentration around 18 months of age. One of the three curves represents the effect of paracetamol on the antibody concentration. The goal of this study is to gain insight in the development of the antibody concentrations after prophylactic and therapeutic paracetamol treatment in adults during vaccination.

Paracetamol is an over the counter drug, and commonly regarded as quite safe, provided the information on the label is followed. It is unlikely, that producers of paracetamol will invest in evaluating potential adverse effects of paracetamol. The potential immune suppressive effects of paracetamol could result in an increasing susceptibility for infections and lower effectiveness of vaccination. Such effects of paracetamol are highly relevant for health authorities who advise on the use of paracetamol as a prophylactic measure for vaccination-induced adverse responses.

# OBJECTIVES

## Primary Objective

The study of Prymula et al. showed the decreased effect of prophylactic paracetamol use during vaccination on the antibody concentrations. Moreover, studies showed that chickenpox in children and rhinovirus infection in adults deteriorates by the use of paracetamol. It is important to get insight in the effects of paracetamol on the vaccination response. In this study we will use hepatitis B vaccination in adults as a model. Hepatitis B is a primary vaccination for participants (students of the Hogeschool Utrecht).

Phase 1: The goal of this study is to investigate possible immune modulating effects of prophylactic paracetamol use on the immune response to hepatitis B vaccination in adults.

It is important to realize that we only start with phase 2 if we find relevant effects of paracetamol on the immune response after vaccination in phase 1.

Phase 2: The goal of the “follow-up” study is to study the association between timing of the use of paracetamol during hepatitis B vaccination and the development of the immune response in adults.

## Secondary Objective

After confirmation of the suppressive effects of paracetamol on the antibody concentration, the underlying mechanisms leading to the reduced immune response will be analysed. Lympohocyte subpopulations, memory B- and T-cells) will be measured.

# STUDY DESIGN

In a prospective cohort, students (≥18 years) of the Hogeschool Utrecht, who are vaccinated with the hepatitis B vaccine as part of their healthcare training, will be included. The students will receive the normal hepatitis B vaccination, three doses at 0 (primary),1 (first booster) and 6 (second booster) months.

The study is composed of two phases. In phase 1, the study participants are randomly assigned to two groups: prophylactic paracetamol group and control group. In phase 2 the study participants are randomly assigned to three groups: prophylactic paracetamol group, therapeutic paracetamol group and control group. The paracetamol treatment consist of three doses of paracetamol administrated orally within the first 24 hours directly (prophylactic) or 6 hours after (therapeutic) the primary and first booster vaccination.

By studying the prophylactic and therapeutic group, the most common situations of paracetamol treatment during vaccination are taking into account. It is known that people use paracetamol prior to vaccination (prophylactic use) to prevent fever and pain after vaccination. Moreover, people start taking paracetamol as symptoms occur after vaccination (therapeutic use). It is known that most symptoms occur within 6-8 hours after vaccination.

The hepatitis B vaccination will be administrated at the Hogeschool Utrecht in October (vaccine 1, primary vaccination), November (vaccine 2, first booster vaccination), March (vaccine 3, second booster vaccination), and blood collection in April. This schedule is according to the normal procedure at the Hogeschool Utrecht. Participants will be assigned to a treatment group and will stay in this study group during the whole vaccination procedure. Participants in the prophylactic and therapeutic group will take paracetamol during the primary and first booster vaccination. During the second booster vaccination none of the participants will take paracetamol. Participants have the responsibility to take paracetamol at the correct times. The normal blood collection takes place one month after the second booster vaccination to measure protective antibody concentrations. Three more tubes will be collected at that moment to measure the functionality of lymphocyte subpopulations. One extra blood collection will take place directly before the second booster vaccination to measure the protective antibody concentrations. More information is available in Capture 7 “Methods”.

# STUDY POPULATION

## Population (base)

A population of healthy young health care students of 18 years or older will be recruited at the Hogeschool Utrecht. The students are routinely vaccinated with hepatitis B vaccine, therefore the vaccination is not an extra load for the participants. Both sexes will be included.

## Inclusion criteria

In order to be eligible to participate in this study, a subject must meet all of the following criteria:

- Participants in good general health.
- Able to perform the study according to the procedures.
- Provision of written informed consent by the participant.

## Exclusion criteria

A potential subject who meets any of the following criteria will be excluded from participation in this study:

- Use of paracetamol, NSAIDs within 24 hours before the vaccinations or blood collection.
- History of acute or chronic hepatitis B.
- Present evidence of serious disease(s) demanding immunosuppressive medical treatment, like corticosteroids that might interfere with the results of the study within 3 months.
- Any known primary or secondary immunodeficiency.
- Other vaccination within one month before the blood collection.
- Allergic reaction to one of the substances of the hepatitis B vaccine.
- In case of a participant is having fever (> 38,5^o^C) within 2 days before the vaccination will take place and before blood sampling , which can interfere with the cellular immune responses at that time, another appointment for the vaccination and/or blood collection will be made.

## Sample size calculation

Phase 1:

In the first part of this study, a prophylactic and control group will be investigated. The results of Prymula et al., are used to calculate the number of participants in this study . The power analysis is based on a difference between the paracetamol group and the control group. Antibody concentrations were used as an outcome measurement and therefore the median values $\nu_{1}=10^{\mu_{1}}$ en $\nu_{2}=10^{\mu_{2}}$were compared in both groups. In this case *μ*_1_ and *μ*_2_ are the mean values of the log10 antibody concentrations. The difference between the two groups is expressed in terms of the ratio between the two medians.

$$1.65=\frac{\nu_{1}}{\nu_{2}}=10^{\mu_{1}-\mu_{2}}$$

Based on the study of Prymula et al., it is possible to say that the median concentration in group 1 (control) is 65% higher than the median concentration in group 2 (prophylactic paracetamol), equivalent to the difference between the mean log concentration in the two groups: $\mu_{1}-\mu_{2}=\log_{10}1.65=0.22$

This decrease in titer is relevant, because Graham et al. showed that adults infected with Rhinovirus who used paracetamol suffer from more symptoms and lower virus clearance (Graham, Burrell et al., 1990). A comparable decrease in antibody concentration was found in the study of Graham et al. compared to the study of Prymula et al. A 65% decrease in antibody titer during Rhinovirus infection in adults causes more symptoms and lower virus clearance.

Prymula et al. showed confidence intervals of the median $\nu=10^{\mu}$ of all different groups. By using the low (*L*) and high (*H*) limits of the confidence intervals and the number of participants (*N),* it is possible to calculate the standard deviation (S) of the logarithm of the antibody concentrations. The standard deviation of the logarithm is used for the calculation of the number of participants.

$$S=\sqrt{n}\frac{\log_{10}(H/L)}{2\times1.96}=0.34$$

A significance level of 0.05 has been taken into account. The expected effect of paracetamol in this study is a negative effect on the antibody concentration after vaccination. The effect is one sided and therefore we will perform an one sided test resulting in a higher power.

$$1-\Phi\left(z_{1-\alpha}-\frac{\mu_{1}-\mu_{2}}{\sigma\sqrt{\frac{1}{n_{1}}+\frac{1}{n_{2}}}}\right)$$

| *Table 1. Number of participants per group* | | |  |  | | |
| --- | --- | --- | --- | --- | --- | --- |
| **65% difference in antibody concentration between the groups** | | |  | **50% difference in antibody concentration between the group** | | |
| **Number of participants** | | **Power of detection** |  | **Number of participants** | | **Power of detection** |
| Group 1 | Group 2 |  |  | Group 1 | Group 2 |  |
| 35 | 35 | 0.85 |  | 35 | 35 | 0.70 |
| 40 | 40 | 0.89 |  | 40 | 40 | 0.75 |
| 45 | 45 | 0.92 |  | 45 | 45 | 0.79 |
| 50 | 50 | 0.94 |  | 50 | 50 | 0.83 |
| 55 | 55 | 0.96 |  | 55 | 55 | 0.86 |
| 60 | 60 | 0.97 |  | 60 | 60 | 0.88 |
| 63 | 63 | 0.97 |  | 63 | 63 | 0.90 |
| 65 | 65 | 0.98 |  | 65 | 65 | 0.90 |

Every year, Hogeschool Utrecht approaches her students, who could be exposed professionally to hepatitis B, to participate in the hepatitis B vaccination procedure. At least 50 participants/group are necessary to reach a power of 0.94. A drop-out of 20% is expected. After including this drop-out percentage in the power analysis, the number of participants /group will be 126 (if 50=80%, than 63 = 100%). Table 1 shows the power after a higher non-expected drop-out. The power is still adequate.

The power analysis is based on the expected 65% difference between the prophylactic and control group, as shown in the Prymula et al study. In the Prymula et al. study, the participants were children and it could be that adults in this study are less sensitive for the immune modulating effect of paracetamol. It is known that the immune response in infants could be substantially different compared to adults. These knowledge is also included in the power analysis by using a 50% difference between the antibody concentrations in the paracetamol and control group. The calculated 63 participants still reach a power of 0.90. A lower immune modulating effect of paracetamol is still good detectable.

All participants could be included in one year. Therefore, all students ≥18 years who participate in the hepatitis B vaccination procedure of the Hogeschool Utrecht (*n*=720) should be approached to participate in this study. 20% of the students should offer their participation.

Phase 2

In Phase 1, an immune modulating effect of prophylactic paracetamol treatment during vaccination was found. Therefore, phase 2 will be executed. In phase 1, a decrease of ±30% (*p*=0.085) in antibody concentration was found in the paracetamol group. The power analysis of phase 2 is based on the results of phase 1.

The data in phase 1 was not normally distributed and therefore a non-parametric test (Mann Whitney U) was used to compare the GMCs of the control- and paracetamol group. This result in a clear effect of paracetamol, however the p-value was not significant. To reach significance, more students should be included. Therefore, phase 2 will be used to investigate the effects of therapeutic paracetamol use, but also to extend the prophylactic and control group (phase 1).

The power analysis in phase 2 is based on a non-parametric test (2 groups = Mann-Whitney, >2 groups = Kruskal-Wallis). Data of the control and prophylactic paracetamol group of phase 1 is used to calculate the number of participants. There are three possible scenarios for the therapeutic paracetamol group; a response comparable to the control group, a response comparable to the prophylactic group or a response between the control and prophylactic group. In case of the third scenario, the effect of paracetamol is ≤ 15%. This effect is too small to induce changes in the advice on the use of paracetamol as a prophylactic measure for vaccination-induced adverse responses. Therefore, the power analysis is based on scenario 1 and 2. We assume that the data distribution in the therapeutic paracetamol group is comparable with the data distribution in the prophylactic paracetamol group.

The power analysis is performed as follows:

- N samples were drawn from the comparable groups to calculate the number of participants N. If need be, the cohort is totally used (i) before a sample was drawn (i+1).
- The drawn data was compared by using an one-sided Mann-Whitney U test (2 groups) or Kruskal-Wallis (3 groups), level of significance is p<0.05.
- 10.000 samples were tested at random and the power calculation was based on the number of significant results in relation to the number of tests.

The power analysis resulted in 140 participants per treatment group to obtain a power of 0.9. A drop-out of 20% is expected based on phase 1. After including this drop-out percentage in the power analysis, the number of participants in total will be 525 (if 140=80%, than 175 = 100%; 175 x 3 treatments = 525).

At the end of both studies, we will combine the data of phase 1 and phase 2. Therefore, the number of participants in the control and prophylactic paracetamol can be reduced by the number of participants that we have included in phase 1. The number of participants in the prophylactic and control group will be 75 participants.

The total number of participants will be: 75 + 75 + 140 = 290 participants + 20% drop-out = 363 participants. This is approximately 50% of the total number of students of Hogeschool Utrecht that will receive the hepatitis B vaccination.

# TREATMENT OF SUBJECTS

## Investigational product/treatment

Paracetamol (acetaminophen) is a commonly used analgesic and antipyretic drug. This over-the-counter drug is available in every supermarket and pharmacy. In this study, paracetamol will be delivered by a pharmacy.

Phase 1: At time of the first and second vaccination, participants in the prophylactic paracetamol group receive the required amount of paracetamol (6 tablets) to take home. Participants have the responsibility to take paracetamol at the correct times.

Phase 2: At time of the first and second vaccination, participants in the prophylactic and therapeutic paracetamol group receive the required amount of paracetamol (6 tablets) to take home. Participants have the responsibility to take paracetamol at the correct times.

Each participant will take three times (every 8 hours) 2 tablets of 500mg paracetamol. This is the maximum allowed dose in the Netherlands (see chapter 6). By using the maximal allowed dose, one can determine with certainty that paracetamol could have an effect on the immune response after vaccination.

By studying the prophylactic and therapeutic group, the most common situations of paracetamol treatment during vaccination are taking into account. It is known that people use paracetamol prior to vaccination (prophylactic use) to prevent fever and pain after vaccination. Moreover, people start taking paracetamol as symptoms occur after vaccination (therapeutic use). It is known that most symptoms occur within 6-8 hours after vaccination.

The paracetamol treatment is approximately 24 hours, resulting in an effective fever and pain control.

In actual practice, a longer paracetamol treatment is not commonly used. Possible effects of longer treatment are not relevant for advice of the common paracetamol treatment during vaccinations.

## Use of co-intervention

It is not desired that participants use paracetamol or anti-inflammatory agents within 48 hours before and/or after vaccination/blood collection. The researchers will inform the participants an extra time during the vaccination procedure. It is important that participants only take the prescribed paracetamol. If it is necessary to use paracetamol or anti-inflammatory agents, the participants should inform the researchers. These participants will be excluded from the study to prevent disruption of the study results.

## Escape medication

One of the adverse events of vaccination is fever. Only when the fever is >39C, participants can use paracetamol to treat this adverse effect. If it is necessary to use paracetamol or anti-inflammatory agents, the participants should inform the researchers. These participants will be excluded from the study to prevent disruption of the study results.

# INVESTIGATIONAL MEDICINAL PRODUCT

Paracetamol (acetaminophen) is an acetanilide derivate with analgesic and antipyretic activity. Paracetamol is used to treat fever and pain caused by flu, cold and after vaccination. Headache, toothache, neuralgia, muscular pain and period pain. Paracetamol is administrated orally or rectally. The recommended dose for adults and children <15 years is 500-1000mg each time with a maximum of 6 tablets / 24 hours.

In this study, paracetamol is administrated orally. The maximum allowed dose is used to study the maximal effect of paracetamol. By using the maximal allowed dose, one can determine with certainty that paracetamol could have an effect on the immune response after vaccination. Paracetamol will be delivered by the pharmacy of Bilthoven. The researchers are responsible for the storage of paracetamol. Paracetamol will be stored at room temperature. Students who participate in the paracetamol groups will receive the required amount of paracetamol (6 tablets) during the first and second vaccination. Relevant information of paracetamol is available in the Summary of Product Characteristics (http://www.medicines.org.uk/emc/medicine/24178/SPC).

Engerix-B (Hepatitis B vaccination) is induced in persons who are not immune as active immunization against hepatitis B-virus infection (HBV). Engerix-B (Hepatitis B vaccinatie) 20 ug (in 1,0 ml suspension) is used intramuscularly in the delta muscle as described in the instruction leaflet for persons < 16 years. The vaccination procedure includes three vaccinations at 0, 1 and 6 months and a measurement of the optimal antibody concentration at 7 months. Engerix-B will be delivered by the pharmacy of UMC Utrecht. Skilled nurses from the UMC Utrecht will administer the vaccination. This is the normal procedure for students who get the vaccination at the Hogeschool Utrecht. The hepatitis B vaccination will be stored at 4°C. Relevant information of Engerix-B (hepatitis B vaccination) is available in the Summary of Product Characteristics (<http://www.medicines.org.uk/emc/medicine/9283/>

SPC/Engerix+B+20+micro grams+1+ml+Suspension+for+injection+in+pre-filled+syringe/).

# METHODS

## Study parameters/endpoints

### Main study parameter/endpoint

Blood samples will be collected in blood collection tubes. Antibody concentrations will be measured at two time points in the plasma of participants.

- Protection against hepatitis B will be determined by using the antibody concentration in the blood one month after the third vaccination.
- The antibody concentration will be measured directly before the third vaccination.

The measurements will give us more information about the influence of paracetamol during the primary vaccination and a possible persistent effect during the booster vaccination. By measuring the antibody concentrations at two time points, it is possible to extrapolate these results to adults that only get primary vaccination. Is the primary vaccination in combination with paracetamol treatment protective enough? The antibody concentration will be measured in phase 1 and 2 at the same time points.

### Secondary study parameters/endpoints

The secondary study parameter will be the kinetics of B-and T-cell memory immune responses against the vaccine proteins of hepatitis B. Blood samples will be collected in vacutainer cell preparation tubes to separate the Peripheral Blood Mononuclear Cells (PBMC’s). PBMC’s will be divided in purified B-cell populations and T-cell populations. B-cells will be cultured and memory B-cells will be polyclonal stimulated. After 5 days, B-cell memory responses will be measured against hepatitis B antigens and tetanus toxoid by ELIspot assays. T-cells will be cultured and T-cell memory responses will be measured against hepatitis B antigens, tetanus toxoid and pokeweed mitogen (PWM) by FACS of the cells and Luminex of the culture supernatants.

## Randomisation, blinding and treatment allocation

Students give their permission to participate in this study by signing an informed consent. A randomization list is made on forehand in SPSS and as a student come in to participate the next place on the list will be allocated to the student.

Phase 1: The control group will not take paracetamol during the vaccinations. The prophylactic paracetamol group will take paracetamol for 24 hours starting directly after the primary and first booster vaccination.

Phase 2: The control group will not take paracetamol during the vaccinations. The prophylactic paracetamol group will take paracetamol for 24 hours starting directly after the primary and first booster vaccination. The therapeutic paracetamol group will take paracetamol for 24 hours starting 6 hours after the primary and first booster vaccination.

In the prophylactic and therapeutic paracetamol group, the participants will take 1000mg paracetamol (2x 500mg tablet) every 8 hours. In the control group, the participants will not take paracetamol or a placebo. The treatment with paracetamol is therefore not blinded and participants are aware of a prophylactic or therapeutic treatment. However, it is not expected that this knowledge could influence the development of antibody concentrations after vaccination.

## Study procedures

This study is conducted of two phases. Phase 1 will start in September 2011 and phase 2 will start in September 2012. It is important to realize that we only start with phase 2 if we find any effects of paracetamol on the immune response after vaccination in phase 1.

Hogeschool Utrecht approaches her students (approximately 900 students, 90% ≥ 18 years), who are professionally exposed to hepatitis B, to participate in the hepatitis B vaccination procedure by e-mail. A couple of days later, the students will receive information from the paracetamol & vaccination study and an informed consent to participate in this study. At the same moment, posters will be attached to bulletin board to make the students familiar with the possibility to participate in this study. In the e-mail and posters, a link to the website will be published to read more about the paracetamol & vaccination study.

Students can register online to participate in this study. In this way, we have an overview of the participants in the study. Students can already read the informed consent on the website or in the e-mail. At the moment of vaccination, the students should sign the informed consent to participate in this study. Students need to be 18 years or older and therefore legally authorized to sign the informed consent.

In case, the number of students who would like to participate is higher than the needed number of students. We will make equally distributed pre selection based on ethnicity, sex and age. Thereafter, the students will be randomly divided in one of the study groups (3. Study design and 7.2 randomization).

Students will receive an e-mail with the time for vaccination. We will inform the participants at the vaccination location about their paracetamol group. The paracetamol will be distributed to the participants in the paracetamol treatment groups.

Students receive the vaccination at three time points, namely 0 month, 1 month and 6 months. The participants in the paracetamol treatment groups will receive paracetamol during the first and second vaccination. None of the participant will receive paracetamol during the third vaccination. Directly before and one month after the third vaccination blood will be drawn to determine the antibody concentrations and functionality of lymphocyte subpopulations (7.1 study endpoints).

## Withdrawal of individual subjects

Subjects can decide not to participate at any time for any reason without any consequences. The investigator can decide to withdraw a subject from the study for urgent medical reasons or after incorrect use of paracetamol or NSAIDs during the study.

## Replacement of individual subjects after withdrawal

If subjects decide to withdraw from the study during the first and second vaccination, new subjects will be included. Students who begin at a later time point with their study will get the first vaccination a couple of months later then the rest of the students. If necessary, we will approach these students to participate in this study.

## Follow-up of subjects withdrawn from treatment

No follow-up

## Premature termination of the study

Hepatitis B and paracetamol are both registered and used in the Netherlands. We do not expect that serious side effects will occur. Therefore we do not expect to premature terminate the study. If need be, the METC will be informed.

##

# SAFETY REPORTING

## Section 10 WMO event

In accordance to section 10, subsection 1, of the WMO, the investigator will inform the subjects and the reviewing accredited METC if anything occurs, on the basis of which it appears that the disadvantages of participation may be significantly greater than was foreseen in the research proposal. The study will be suspended pending further review by the accredited METC, except insofar as suspension would jeopardize the subjects’ health. The investigator will take care that all subjects are kept informed.

## AEs, SAEs and SUSARs

### Adverse events (AEs)

Adverse events are defined as any undesirable experience occurring to a subject during the study, whether or not considered related to the investigational product. All adverse events reported spontaneously by the subject or observed by the investiga­tor or his staff will be recorded.

### Serious adverse events (SAEs)

A serious adverse event is any untoward medical occurrence or effect that at any dose:

- results in death;
- is life threatening (at the time of the event);
- requires hospitalisation or prolongation of existing inpatients’ hospitalisation;
- results in persistent or significant disability or incapacity;
- is a congenital anomaly or birth defect;
- Any other important medical event that may not result in death, be life threatening, or require hospitalization, may be considered a serious adverse experience when, based upon appropriate medical judgement, the event may jeopardize the subject or may require an intervention to prevent one of the outcomes listed above.

The sponsor will report the SAEs through the web portal *ToetsingOnline* to the accredited METC that approved the protocol, within 15 days after the sponsor has first knowledge of the serious adverse events.

SAEs that result in death or are life threatening should be reported expedited. The expedited reporting will occur not later than 7 days after the responsible investigator has first knowledge of the adverse event. This is for a preliminary report with another 8 days for completion of the report.

### Suspected unexpected serious adverse reactions (SUSARs)

Adverse reactions are all untoward and unintended responses to an investigational product related to any dose administered.

Unexpected adverse reactions are SUSARs if the following three conditions are met:

1. the event must be serious (see chapter 9.2.2);
2. there must be a certain degree of probability that the event is a harmful and an undesirable reaction to the medicinal product under investigation, regardless of the administered dose;
3. the adverse reaction must be unexpected, that is to say, the nature and severity of the adverse reaction are not in agreement with the product information as recorded in:

- Summary of Product Characteristics (SPC) for an authorised medicinal product;
- Investigator’s Brochure for an unauthorised medicinal product.

The sponsor will report expedited the following SUSARs through the web portal *ToetsingOnline* to the METC:

- SUSARs that have arisen in the clinical trial that was assessed by the METC;
- SUSARs that have arisen in other clinical trials of the same sponsor and with the same medicinal product, and that could have consequences for the safety of the subjects involved in the clinical trial that was assessed by the METC.

The remaining SUSARs are recorded in an overview list (line-listing) that will be submitted once every half year to the METC. This line-listing provides an overview of all SUSARs from the study medicine, accompanied by a brief report highlighting the main points of concern.

The expedited reporting of SUSARs through the web portal ToetsingOnline is sufficient as notification to the competent authority.

The sponsor will report expedited all SUSARs to the competent authorities in other Member States, according to the requirements of the Member States.

The expedited reporting will occur not later than 15 days after the sponsor has first knowledge of the adverse reactions. For fatal or life threatening cases the term will be maximal 7 days for a preliminary report with another 8 days for completion of the report.

## Annual safety report

In addition to the expedited reporting of SUSARs, the sponsor will submit, once a year throughout the clinical trial, a safety report to the accredited METC, competent authority, and competent authorities of the concerned Member States.

This safety report consists of:

- a list of all suspected (unexpected or expected) serious adverse reactions, along with an aggregated summary table of all reported serious adverse reactions, ordered by organ system, per study;
- a report concerning the safety of the subjects, consisting of a complete safety analysis and an evaluation of the balance between the efficacy and the harmfulness of the medicine under investigation.

## Follow-up of adverse events

All AEs will be followed until they have abated, or until a stable situation has been reached. Depending on the event, follow up may require additional tests or medical procedures as indicated, and/or referral to the general physician or a medical specialist.

SAEs need to be reported till end of study within the Netherlands, as defined in the protocol

## Data Safety Monitoring Board (DSMB)

This study is categorized as a low risk study, therefore a minimum level of monitoring is necessary. In this study, an independent research nurse of the RIVM (DSMB) will monitor the study. The name of this GCP qualified nurse is Anneke Westerhof (030 274 4166). In the schedule below, the task and responsibility of this person is described. The visits of the DSMB will take places before, during and at the end of the study.

The advice(s) of the DSMB will only be sent to the sponsor of the study. Should the sponsor decide not to fully implement the advice of the DSMB, the sponsor will send the advice to the reviewing METC, including a note to substantiate why (part of) the advice of the DSMB will not be followed.

Phase 1

| **Monitor frequencies** | 3 visits |
| --- | --- |
| **Patients** | Inclusion tempo and withdrawal percentage |
| **Investigator File** | Presence and completeness of the investigator file |
| **Informed Consent** | 50% of the study population (126 participants) |
| **In-/exclusion criteria** | First 3 participants, thereafter 10% of the other participants. If one of the participants is included and doesn’t meet the inclusion criteria, all participants will be examined. |
| **Source Data verification** | 10%. Primary endpoint is the antibody concentration of the participant. Variables will be discussed during the initiation visit. |
| **SAEs en SUSARs** | 10% of the participants on possible missed SAEs + 100% verification of reported SAEs and SUSARs. If one of the reported SAEs or SUSARs is incorrect, all reports will be examined. |
| **Study medication** | Examine the instructions for participants. |
| **Study procedures, equipment and facilities** | Examine the instruction for execution of the study and examine training of the study personnel. |
| **Laboratory & pharmacy** | Verify the GLP/GMP certification of the laboratory and pharmacy. |

Phase 2

| **Monitor frequencies** | 3 visits |
| --- | --- |
| **Patients** | Inclusion tempo and withdrawal percentage |
| **Investigator File** | Presence and completeness of the investigator file |
| **Informed Consent** | 25% of the study population (252 participants) |
| **In-/exclusion criteria** | First 3 participants, thereafter 10% of the other participants. If one of the participants is included and doesn’t meet the inclusion criteria, all participants will be examined. |
| **Source Data verification** | 10%. Primary endpoint is the antibody concentration of the participant. Variables will be discussed during the initiation visit. |
| **SAEs en SUSARs** | 10% of the participants on possible missed SAEs + 100% verification of reported SAEs and SUSARs. If one of the reported SAEs or SUSARs is incorrect, all reports will be examined. |
| **Study medication** | Examine the instructions for participants. |
| **Study procedures, equipment and facilities** | Examine the instruction for execution of the study and examine training of the study personnel. |
| **Laboratory & pharmacy** | Verify the GLP/GMP certification of the laboratory and pharmacy. |

# STATISTICAL ANALYSIS

## Descriptive statistics

*Antibody concentrations*

Phase 1: Antibody concentrations against hepatitis B will be determined in the prophylactic paracetamol group and control group at two time points, directly before and one month after the third vaccination.

Phase 2: Antibody concentrations against hepatitis B will be determined in the prophylactic paracetamol group, therapeutic paracetamol group and control group at two time points, directly before and one month after the third vaccination.

The mean antibody concentration per treatment group per time point will be calculated as geometric mean concentration (GMC) with 95% confidence interval. The GMC will be calculated by using the following formula:

*Functionality of specific lymphocyte subpopulations*

Functionality of lymphocyte subpopulations, such as regulatoir T-cells, T-helper1 cells and T-helper2 cells, will be determined in the prophylactic paracetamol group and control group (phase 1) and in the prophylactic paracetamol group, therapeutic paracetamol group and control group (phase 2). Functionality, including proliferation and cytokine production, will be determined one month after the third vaccination. Outcomes will be calculated as GMC with 95% confidence interval.

## Univariate analysis

*Antibody concentrations*

Phase 1: The GMC of the control group will be compared to the GMC of the prophylactic paracetamol group. In the study of Prymula et al. is demonstrated in children that the antibody concentrations in the paracetamol group are lower compared to the control group. In this study, we expect an effect in the same direction; a lower antibody concentration in the prophylactic paracetamol group. Significance difference in GMC of the control compared to the prophylactic group will be calculated by using the Student’s T-test (‘unpaired samples, one-tailed’).

Phase 2: The GMC of the therapeutic paracetamol group will be compared to the GMC of the prophylactic and control group. Significance difference in GMC of the control group and the GMC of the treatment groups will be calculated by using ANOVA and post-hoc analyses.

In both phases of this study, the effect of the second booster vaccination on the antibody concentrations will be studied. The GMCs of the two time points within one study group will be compared. A ratio between these two values can be determined for each study group.

Phase 1: Significance difference in ratio of the control group and prophylactic paracetamol group will be calculated by using Student’s t-test. This outcome can show a possible difference in the power of the second booster vaccination in the control group compared to the prophylactic paracetamol group.

Phase 2: Significance difference in ratio of the control group, prophylactic paracetamol group or therapeutic paracetamol group will be calculated by using ANOVA and post-hoc analyses. This outcome can show a possible difference in the power of the second booster vaccination in the control group compared to the prophylactic paracetamol group or therapeutic paracetamol group.

*Functionality of specific lymphocyte subpopulations*

Phase 1: Significance difference in outcome measurements (proliferation, production of IL-2, IFNγ, and IL-4) of the functionality will be calculated by using the Student’s t-test (‘unpaired samples, two-tailed).

Fase 2: Significance difference in outcome measurements (proliferation, production of IL-2, IFNγ, and IL-4) of the functionality will be calculated by using ANOVA and post-hoc analyses.

In both phases of this study, the effect of the second booster vaccination on the lymphocyte subpopulations will be studied. The GMCs of the two time points of the outcome measurements of functionality within one study group will be compared. A ratio between these two values can be determined for each outcome measurement within one study group.

Phase 1: Significance difference in ratio of the control group and prophylactic paracetamol group will be calculated by using Student’s t-test. This outcome can show a possible difference in effect of the second booster vaccination on the outcome measurements in the control group compared to the prophylactic paracetamol group.

Phase 2: Significance difference in ratio of the control group, prophylactic paracetamol group or therapeutic paracetamol group will be calculated by using ANOVA and post-hoc analyses. This outcome can show a possible difference in effect of the second booster vaccination on the outcome measurements in the control group compared to the prophylactic paracetamol group or therapeutic paracetamol group.

Normal distribution of the data (antibody concentrations and functionality of specific subpopulations) will be calculated by using T-tests (equality of means), F-test (equality of variances), and Kolmogorv-Smirnov-test (normal distribution).

It could be that the data is not normally distributed; subsequently non-parametric tests (Mann-Whitney or Kruskal-Wallis) will be used to calculate significance differences.

# ETHICAL CONSIDERATIONS

## Regulation statement

This study will be conducted according to the principles of the Declaration of Helsinki (Amendement of Seoul 2008, see for the most recent version: [www.wma.net](http://www.wma.net/)) and in accordance with the Medical Research Involving Human Subjects Act (WMO).

## Recruitment and consent

Hogeschool Utrecht approaches her students (approximately 800 persons, 90% ≥ 18 years), who could be exposed professionally to hepatitis B, by e-mail to participate in the hepatitis B vaccination procedure. A couple of days later, the students will receive information from the paracetamol & vaccination study and an informed consent to participate in this study by e-mail. At the same moment, posters will be attached to bulletin board to make the students familiar with the possibility to participate in this study. In the e-mail and posters, a link to the website will be published to read more about the paracetamol & vaccination study.

Students can register online to participate in this study. In this way, we have an overview of the participants in the study. Students can already read the informed consent on the website or in the e-mail. At the moment of vaccination, the students should sign the informed consent to participate in this study. After registration, the investigators can estimate the number of participants and make the randomization list.

Students will receive an e-mail with the time for vaccination. We will inform the participants at the vaccination location about their treatment group. Phase 1: prophylactic treatment or control group. Phase 2: prophylactic, therapeutic or control group.

## Benefits and risks assessment, group relatedness

Paracetamol is commonly used during vaccination. There are several indications that paracetamol has an immune modulating effect. This effect could affect the vaccination response. It is very important to obtain more information about the immune reactions after vaccination in combination with paracetamol treatment. Particularly, the effects of paracetamol on the primary vaccination in adults are not known. This study will demonstrate the effects of paracetamol on the vaccination response in adults and also investigate the role of timing of paracetamol in this effect on vaccination response. Participation to this study will significantly contribute to the scientific knowledge, resulting in a better advice of health organizations about the use of paracetamol during vaccination procedures. This advice could be extrapolate to vaccinations that are used for travelling, this is also a primary contact with the antigen.

Potentially, the results of this study could be used as a result for infants. At this moment, the effects of therapeutic paracetamol use on the vaccination response in infants are not clear, while therapeutic paracetamol use is frequently used in this group. In the Netherlands, most vaccinations are given to infants.

## Compensation for injury

The sponsor of this study obtained a release from the METC Utrecht to get a liability insurance. The committee presumed that this study is not hazardous for the study subjects.

## Incentives

A small compensation, an iris-cheque at the value of 15 euro, will be given to the participants.

# ADMINISTRATIVE ASPECTS, MONITORING AND PUBLICATION

## Handling and storage of data and documents

Data of study participant will be coded; every participant will get a study number. The Principal Clinical Investigator assures that the anonymity of the participants is maintained; keeping separate log of codes, names and addresses of participants. During the study blood will be stored at the RIVM Bilthoven. 15 years after the study all materials will be destroyed, on request of the participants the material can be destroyed earlier.

## Amendments

A ‘substantial amendment’ is defined as an amendment to the terms of the METC application, or to the protocol or any other supporting documentation, that is likely to affect to a significant degree:

- the safety or physical or mental integrity of the subjects of the trial;
- the scientific value of the trial;
- the conduct or management of the trial; or
- the quality or safety of any intervention used in the trial.

All substantial amendments will be notified to the METC. Non-substantial amendments will not be notified to the accredited METC and the competent authority, but will be recorded and filed by the sponsor. This is the first amendment of this study. The changes are:

- In phase 2, four study groups were described (control, prophylactic and two different therapeutic paracetamol groups). This number will be reduced to three study groups (control, prophylactic and therapeutic paracetamol groups).
- The power calculation of phase 2 is calculated again by using results from phase 1.
- In the study protocol, two blood drawings are described for phase 1 and phase 2. In phase 2 we will draw blood at the same time points, but we will draw a different total volume of blood.

## Annual progress report

The sponsor/investigator will submit a summary of the progress of the trial to the accredited METC once a year. Information will be provided on the date of inclusion of the first subject, numbers of subjects included and numbers of subjects that have completed the trial, serious adverse events/ serious adverse reactions, other problems, and amendments.

## End of study report

The investigator will notify the accredited METC of the end of the study within a period of 8 weeks [90 days]. The end of the study is defined as the last patient’s last visit.
In case the study is ended prematurely, the investigator will notify the accredited METC, including the reasons for the premature termination. Within one year after the end of the study, the investigator/sponsor will submit a final study report with the results of the study, including any publications/abstracts of the study, to the accredited METC.

## Public disclosure and publication policy

The coordinating investigators will submit the results of the study for publication in collaboration with the principal investigators.

# REFERENCES

Baken, K. A., J. L. Pennings, et al. (2008). "Overlapping gene expression profiles of model compounds provide opportunities for immunotoxicity screening." Toxicol Appl Pharmacol **226**(1): 46-59.

David S, V.-d. B. P., Van der Maas NAT (2009). "Paracetamol for adverse events after pertussis immunisation in infancy in the netherlands." Poster presentation Espid.

Doran, T. F., C. De Angelis, et al. (1989). "Acetaminophen: more harm than good for chickenpox?" J Pediatr **114**(6): 1045-1048.

Farquhar, H., J. Crane, et al. (2009). "The acetaminophen and asthma hypothesis 10 years on: A case to answer." J Allergy Clin Immunol **124**(4): 649-651.

Graham, N. M., C. J. Burrell, et al. (1990). "Adverse effects of aspirin, acetaminophen, and ibuprofen on immune function, viral shedding, and clinical status in rhinovirus-infected volunteers." J Infect Dis **162**(6): 1277-1282.

Gross, P. A., R. A. Levandowski, et al. (1994). "Vaccine immune response and side effects with the use of acetaminophen with influenza vaccine." Clin Diagn Lab Immunol **1**(2): 134-138.

Jackson, L. A., M. Dunstan, et al. (2006). "Prophylaxis with acetaminophen or ibuprofen for prevention of local reactions to the fifth diphtheria-tetanus toxoids-acellular pertussis vaccination: a randomized, controlled trial." Pediatrics **117**(3): 620-625.

Koniman, R., Y. H. Chan, et al. (2007). "A matched patient-sibling study on the usage of paracetamol and the subsequent development of allergy and asthma." Pediatr Allergy Immunol **18**(2): 128-134.

Manley, J. and A. Taddio (2007). "Acetaminophen and ibuprofen for prevention of adverse reactions associated with childhood immunization." Ann Pharmacother **41**(7): 1227-1232.

Prymula, R., C. A. Siegrist, et al. (2009). "Effect of prophylactic paracetamol administration at time of vaccination on febrile reactions and antibody responses in children: two open-label, randomised controlled trials." Lancet **374**(9698): 1339-1350.

Rebordosa, C., M. Kogevinas, et al. (2008). "Pre-natal exposure to paracetamol and risk of wheezing and asthma in children: a birth cohort study." Int J Epidemiol **37**(3): 583-590.

Shaheen, S. O., J. A. Sterne, et al. (2000). "Frequent paracetamol use and asthma in adults." Thorax **55**(4): 266-270.

Toma, T. (2000). "High paracetamol intake may be linked with asthma." BMJ **321**(7270): 1178A.

Ueno, K., K. Yamaura, et al. (2000). "Acetaminophen-induced immunosuppression associated with hepatotoxicity in mice." Res Commun Mol Pathol Pharmacol **108**(3-4): 237-251.

Yamaura, K., K. Ogawa, et al. (2002). "Inhibition of the antibody production by acetaminophen independent of liver injury in mice." Biol Pharm Bull **25**(2): 201-205.
